# Effects of different exercise types on visceral adipose tissue and glycemic parameters in adults with stage 1 cardiovascular-kidney-metabolic syndrome: a systematic review and network meta-analysis of randomized controlled trials

**DOI:** 10.3389/fcvm.2026.1873140

**Published:** 2026-09-29

**Authors:** Xinda Wei, Hanyu Zhao, Shuang Yue, Tao Ren, Yuhang Yang, Jiazhen Wang, Ruihua Cao, Ji Fang, Fan Su, Fei Zhong

**Affiliations:** 1 College of Physical Education, China Three Gorges University, Yichang, China; 2 Institute of Sports Rehabilitation, China Three Gorges University, Yichang, China

**Keywords:** cardiovascular-kidney-metabolic syndrome, exercise training, fasting blood glucose, glycated hemoglobin, visceral adipose tissue

## Abstract

**Background:**

Cardiovascular-kidney-metabolic (CKM) syndrome, particularly at stage 1, is increasingly prevalent worldwide. Although exercise interventions are recognized as effective in reducing visceral adipose tissue (VAT) and improving glucose metabolism, their effects in adults with stage 1 CKM syndrome remain unclear. This study aimed to systematically analyze the effects of different exercise types on VAT, fasting blood glucose (FBG), and glycated hemoglobin (HbA1c) in adults with stage 1 CKM syndrome.

**Methods:**

We searched six bibliographic databases—China National Knowledge Infrastructure (CNKI), WanFang Database, VIP Database, Web of Science, Embase, and PubMed—from inception to 15 April 2026, using the PICOS framework. A frequentist random-effects Network Meta-Analyses was conducted using STATA 18.0 software to evaluate the effects of interventions on VAT, FBG and HbA1c. The effectiveness of various exercise intervention types was ranked by calculating the surface under the cumulative ranking curve (SUCRA), and the risk of bias was evaluated using the Cochrane Risk of Bias (ROB 2.0) tool. This study protocol was registered in PROSPERO (CRD420261304633).

**Results:**

Thirty-seven randomized controlled trials involving 3483 participants were included. Compared with the control group, aerobic training significantly decreased VAT [standardized mean difference = −0.44, 95% confidence interval (CI): (−0.62, −0.26)], while mind–body exercise (MBE) significantly improved FBG [mean difference (MD) = −1.16 mmol/L, 95% CI: (−1.57, −0.75)] and HbA1c [MD = −0.35%, 95% CI: (−0.62, −0.07)]. Subgroup analysis revealed that high-intensity interval training with an intervention duration of ≤ 12 weeks (SUCRA = 67%) and aerobic training with a duration of >12 weeks (SUCRA = 94.9%) had the highest probability of being the most effective intervention for reducing VAT in included studies. In addition, mind–body exercise ranked highest for FBG improvement, though with limited certainty.

**Conclusions:**

These findings provide evidence that different exercise types exert distinct benefits on metabolic health in adults with stage 1 CKM syndrome. In particular, aerobic training may be more effective than other exercise types in reducing VAT, while MBE may contribute to more benefits in improving FBG and HbA1c.

**Systematic Review Registration:**

https://www.crd.york.ac.uk/PROSPERO/view/CRD420261304633, Identifier CRD420261304633.

## 1 Introduction

Cardiovascular-kidney-metabolic (CKM) syndrome is a spectrum of multiorgan disorders arising from pathophysiological interplay among metabolic risk factors, and has been proposed as a disease staging framework by the American Heart Association (1, 2). CKM syndrome is classified into stages 0–4, representing progression in individuals from a healthy state to clinical cardiovascular disease (CVD). Stage 1 of CKM syndrome is characterized by excess adiposity, dysglycemia, or both, encompassing individuals with overweight/obesity or prediabetes (2). Previous studies have estimated that stage 1 CKM syndrome affects approximately 28% of US adults (3, 4) and 26% of the Chinese population (5). These adults with stage 1 CKM syndrome may progress to more severe disease states without intervention, presenting as clinical hypertension, hyperglycemia, CVD, and chronic kidney disease. This progression will not only affect their quality of life but also impose a substantial economic burden on individuals, families, and society (2).

**Figure 1:**
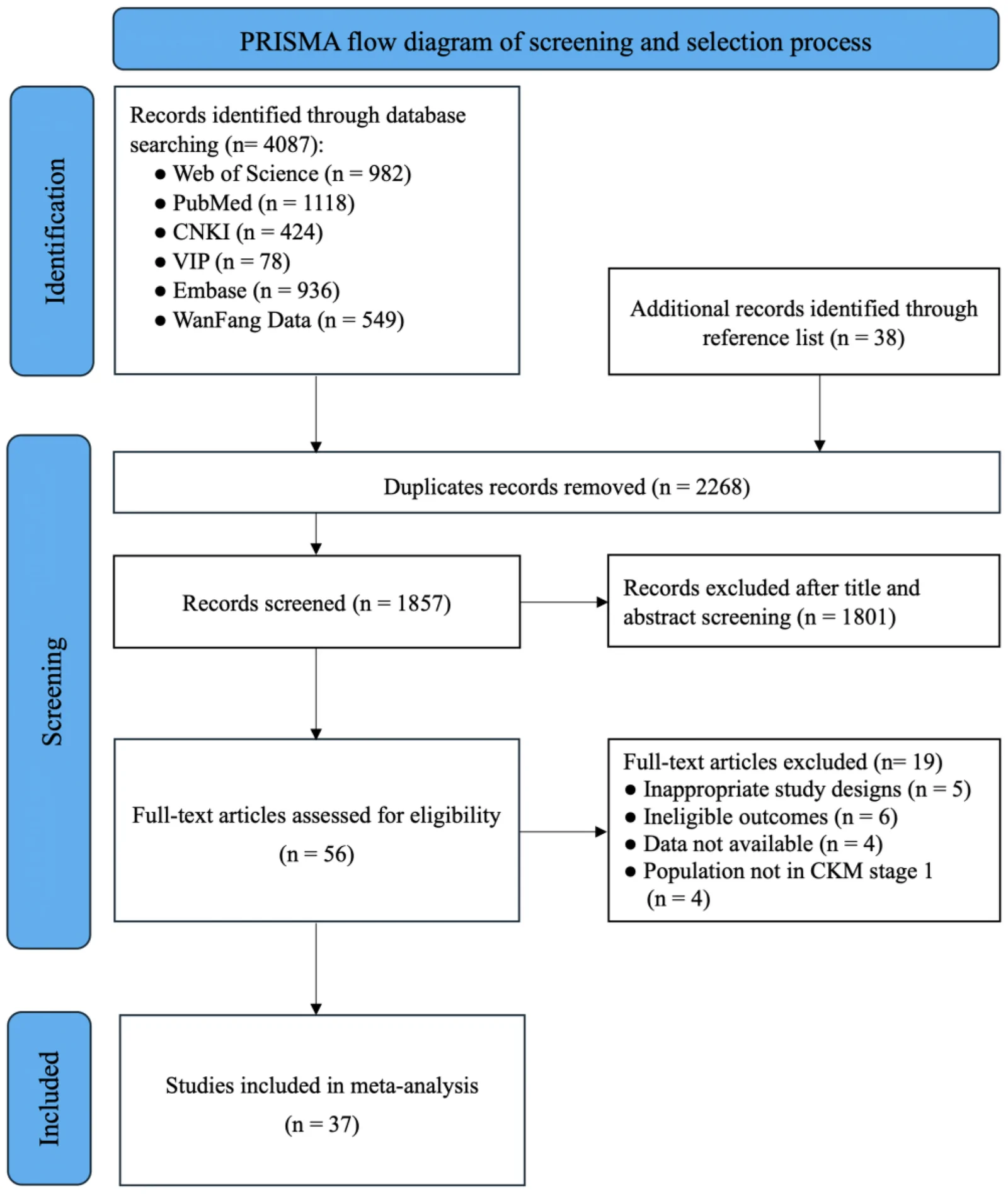
PRISMA flow diagram of study selection.

Visceral adipose tissue (VAT) is considered an early predictor of prediabetes (6, 7). Previous studies have indicated that reduction in VAT was significantly associated with improvement in fasting blood glucose (FBG) levels compared with reduction in subcutaneous fat for subjects with obesity (8). Therefore, individuals with stage 1 CKM syndrome may achieve a more favorable metabolic profile through scientific interventions targeting VAT reduction. Although previous evidence showed that excess adiposity or dysglycemia in stage 1 CKM syndrome can be improved by therapeutic interventions such as metabolic surgery and medications (9, 10), these interventions may induce an increased risk of accidental death (11) and gastrointestinal symptoms (12). Notably, growing evidence supports regular exercise as a non-medical intervention that can lead to significant improvements in human health (13, 14), particularly by reducing VAT and improving glucose regulation among obese and diabetic adults (15, 16).

However, existing randomized controlled trials (RCTs) of different types of exercise interventions have yielded inconsistent evidence regarding the effects on VAT, FBG, and glycated hemoglobin (HbA1c) in adults with stage 1 CKM syndrome (17–22). More specifically, combined aerobic and resistance training (CT) or aerobic training alone has demonstrated benefits in improving FBG in some studies (7, 18). However, other studies have failed to show significant effects (19, 23). To our knowledge, the effects of different exercise types on health outcomes can be evaluated using the surface under the cumulative ranking curve (SUCRA) within a network meta-analysis (24). Therefore, this systematic review and network meta-analysis aims to compare the relative efficacy of different exercise types on VAT and FBG levels in adults with stage 1 CKM syndrome. The results of this study not only provide evidence-based rankings of different exercise types to guide clinical practice but also offer valuable insights for improving metabolic health through exercise interventions.

## 2 Methods

### 2.1 Protocol and registration

This study was conducted in accordance with Preferred Reporting Items for Systematic Reviews and Meta-Analyses for Network Meta-Analyses (PRISMA-NMA) guidelines (25). The study protocol was registered in PROSPERO (CRD420261304633).

### 2.2 Inclusion and exclusion criteria

Studies were eligible if they met the following criteria: ① the studies were RCTs; ② the adults were characterized by overweight/obesity [body mass index (BMI) ≥25 or ≥23 kg/m^2^ for Asian ancestry, waist circumference ≥88/102 cm in women/men or ≥80/90 cm for Asian ancestry], and/or prediabetes (FBG ≥100–125 mg/dL or HbA1c between 5.7% and 6.4%) (26); ③ interventions included aerobic training (AT), resistance training (RT), combined aerobic and resistance training (CT), high-intensity interval training (HIIT), and mind–body exercise (MBE); and ④ the primary outcomes included VAT, FBG, and HbA1c. Studies were excluded if the fulfilled the following criteria: ① animal models were included; ② the subjects were adults with other diseases, such as hypertension, diabetes, dyslipidemia, and mental health disorder; or ③ the outcomes of studies were unavailable or incomplete.

### 2.3 Literature search strategy

We searched CNKI, WanFang Database, VIP Database, Web of Science, Embase, and PubMed from inception to 15 April 2026, based on the PICOS framework. (P) Population: adults with stage 1 CKM syndrome; (I) Intervention: exercise training; (C) Comparator: no exercise intervention or low-intensity active control; (O) Outcomes: VAT, FBG, and HbA1c; (S) Study type: RCTs. In addition, the reference lists of published reviews and meta-analyses were screened to identify additional RCTs that met the inclusion criteria. A detailed search strategy is provided in Supplementary Appendix 1. Definitions of the exercise interventions are provided in Supplementary Appendix 2.

### 2.4 Data extraction

Data extraction was performed independently by two researchers (XW and HZ); when disagreements could not be resolved, a third author (FZ) was consulted. As presented in Table 1, the extracted data included study characteristics (author, publication year), sample characteristics (sample size and age), intervention protocols (exercise frequency, duration, type, and cycle), and outcome measures (VAT, FBG, and HbA1c).

**Table 1:** Characteristics of the included studies.

Research, year	Study design	Participant characteristics	Mean age (years)	Exercise intervention	Sample size	Exercise frequency (days/week)	Exercise duration (min)	Exercise period (weeks)	Outcome measures
Guo et al. (39), 2021	RCT	Obesity	20.0 (median)	AT CON	60 60	5 –	60 –	12	①
Lin et al. (40), 2016	RCT	Obesity	20.2	HIIT CON	18 18	2 –	38 –	12	①
Dai et al. (41), 2014	RCT	Obesity	26.2	HIIT CON	10 10	4 –	– –	12	①
Qi et al. (42), 2020	RCT	Obesity	19.0 (median)	CT AT	30 30	3–4	40 40	12	①
Tong et al. (43), 2018	RCT	Obesity	21.0	HIIT CON	16 14	3–4 –	– –	12	①
Chiang et al. (44), 2019	RCT	Obesity	20.1	AT CON	11 9	3 –	30 –	8	①
Ross et al. (45), 2004	RCT	Obesity	43.4	AT CON	17 10	7 –	64 –	14	①②
Haganes et al. (22), 2022	RCT	Overweight/obesity	35.7	HIIT CON	33 33	3 –	32 or 38 –	7	①②③
Friedenreich et al. (46), 2011	RCT	Overweight/obesity	60.9	AT CON	160 160	5 –	45 –	52	①
Nunes et al. (21), 2019	RCT	Obesity	62.6 (median)	CT HIIT	13 13	3	60 28	12	①
Ballin et al. (20), 2019	RCT	Obesity	70.7	HIIT CON	38 39	3 –	18–36 –	10	①
Keating et al. (47), 2017	RCT	Overweight/obesity	44.8	RT CON	15 14	3 –	30–60 –	8	①
Han et al. (48), 2022	RCT	Prediabetes	42.6	CT CON	60 60	3 –	60 –	12	②
Wang et al. (49), 2013	RCT	Prediabetes	51.3	AT CON	70 20	7 –	60 –	12	②
Wu et al. (50), 2018	RCT	Prediabetes	63.0	AT RT	25 25	4	30–40	12	②
Yan et al. (51), 2019	RCT	Prediabetes	62.2	AT RT CON	35 35 35	3 3 –	60 50 –	52	①②③
Jadhav et al. (7), 2024	RCT	Prediabetes	48.9	CT CON	79 79	2–3 –	75 or 150 –	24	①②③
Luo et al. (18), 2023	RCT	Prediabetes	51.0	AT RT CON	26 27 27	3 3 –	50 50 –	12	②
Karaman et al. (52), 2026	RCT	Prediabetes	45.0 (median)	MBE CON	25 24	3 –	60 –	16	②③
Chen et al. (17), 2025	RCT	Prediabetes	52.0 (median)	HIIT AT	33 38	3	30 60	12	①②③
Morey et al. (19), 2012	RCT	Prediabetes with overweight/obesity	67.3	CT CON	180 122	AT ≥ 5 and RT = 3 –	AT ≥ 30 and RT = 15 –	52	②③
Herzig et al. (53), 2014	RCT	Prediabetes with overweight/obesity	58.8	AT CON	38 40	3 –	60 –	12	①②
Hafeez et al. (54), 2025	RCT	Prediabetes with overweight/obesity	31.2	CT CON	84 42	CT = 2 and AT = 1 –	60 –	16	②③
Irwin et al. (55), 2003	RCT	Overweight/obesity	60.8	CT CON	87 86	5 –	≥45 –	52	①
Chow et al. (23), 2021	RCT	Obesity	19.7	AT CON	24 12	3 –	8–43 –	12	②
Chen et al. (56), 2021	RCT	Prediabetes	60.5	AT RT CON	83 82 83	3 3 –	60 50 –	104	②③
Bouchonville et al. (57), 2014	RCT	Obesity	69.5	CT CON	26 27	3 –	90 –	52	①②
Tomeleri et al. (58), 2016	RCT	Obesity	68.2	RT CON	19 19	3 –	45–50 –	8	②
Streb et al. (59), 2021	RCT	Obesity	34.7	CT CON	46 23	3 –	60 –	16	②
Ratajczak et al. (60), 2024	RCT	Overweight/obesity	35.0 (median)	CT CON	28 14	3 –	33 –	12	①②
Yuan et al. (61), 2020	RCT	Prediabetes	60.5	AT RT CON	83 82 83	3 3 –	60 50 –	24	②③
Sawangwong et al. (62), 2023	RCT	Prediabetes	44.0 (median)	MBE CON	32 32	3 –	60 –	12	②③
Siu et al. (63), 2026	RCT	Overweight/obesity	48.2	HIIT CON	210 105	1 or 3 –	25 or 75 –	16	②
Shahiddoust et al. (64), 2025	RCT	Overweight/obesity	34.0 (median)	HIIT CT CON	11 11 11	3 3 –	– – –	8	②
Ho et al. (65), 2012	RCT	Overweight/obesity	53.0	AT RT CT CON	15 16 17 16	3 or 5 3 or 5 3 or 5 –	30 30 30 –	12	②
De Strijcker et al. (66), 2018	RCT	Overweight/obesity	46.5	AT HIIT	8 8	2	40	10	②
Tan et al. (67), 2025	RCT	Overweight/obesity	56.7	HIIT CON	12 12	3 –	20 –	8	①

AT, aerobic training; RT, resistance training; CT, AT + RT; HIIT, high-intensity interval training; MBE, mind–body exercise; CON, control group; ①, VAT; ②, FBG; ③, HbA1c.

### 2.5 Risk of bias assessment

Two researchers (XW and HZ) independently assessed the risk of bias for the included studies using the Cochrane Risk of Bias tool (RoB 2) (27). The assessment covered five domains: randomization process, deviations from intended interventions, missing outcome data, outcome measurement, and selection of the reported result. The overall risk of bias was judged as “low” if all five domains were rated as low risk, as “some concerns” if no domain was at high risk but at least one domain raised some concerns, and as “high” if any domain was rated as high risk. If any discrepancies arose during the quality ratings, a third reviewer (FS) was consulted.

### 2.6 Quality of evidence

The Confidence in Network Meta-Analysis (CINeMA) framework was used to evaluate the certainty of evidence, based on the Grading of Recommendations, Assessment, Development, and Evaluation (GRADE) criteria in this study (28, 29). Six domains were considered in this evaluation: within-study bias, reporting bias, indirectness, imprecision, heterogeneity, and incoherence. The judgments were summarized, and the overall confidence was rated as high, moderate, low, or very low.

### 2.7 Data synthesis and statistical analyses

A random-effects model was used to pool effect estimates of pre- to post intervention changes in both the exercise and control groups across the included studies. Missing standard deviations (SDs) were calculated from standard error or 95% confidence interval (CI) (Supplementary Appendix 3.1). To ensure data independence in the network meta-analysis, multiple groups of similar interventions were pooled into a single node (30, 31) (Supplementary Appendix 3.2).

The SD of the pre–post changes was estimated using the formula based on the Cochrane Handbook for Systematic Reviews of Interventions, with the correlation coefficient “*r*” between baseline and final measurements assumed to be 0.5 (30):

$$\mathrm{SD}\;\mathrm{change}=\sqrt{(SD^{2}\mathrm{pre}+SD^{2}\mathrm{post}\;\text{-}\;2\times r\;\times \mathrm{SDpre}\times \mathrm{SDpost})\;}$$

The pairwise meta-analyses were performed using Review Manager 5.4 (Nordic Cochrane Centre, Denmark) to estimate the effects of exercise training versus control groups (32). The heterogeneity was considered substantial if *I²* exceeded 50% or a *p* value was ≤ 0.10, and a random-effects model was used for analysis (33). Due to the variability in measurement tools and units across studies, the standardized mean difference (SMD) with 95% confidence intervals (CIs) was used for VAT. The mean difference (MD) was used for FBG and HbA1c (Supplementary Appendix 4).

Stata 18.0 software (Stata Corp, College Station, TX, USA) was used to perform a random-effects NMA model (34) within a frequentist framework, following the PRISMA-NMA guidelines (25). The SMD was used to estimate the effect size for VAT, and MD was used for FBG and HbA1c. The pooled effect was presented with 95% (CIs), and the 95% prediction interval was calculated (Supplementary Appendix 5). Sensitivity analysis was performed by excluding individual studies to confirm the robustness of findings. In addition, subgroup analyses were conducted by age and exercise duration.

Inconsistency in the evidence for outcomes was examined by using loop-specific heterogeneity estimates, node-splitting, and design-by-treatment (35) (Supplementary Appendix 6). When the *p-*value was greater than 0.05 for inconsistency, the consistency model was applied (36). To evaluate the transitivity assumption, distribution of baseline characteristics was assessed across the included studies. Between-study heterogeneity was assessed using the variance (*τ*^2^). SUCRA was used to rank the effects on different types of exercises (37). SUCRA values range from 0% to 100%, with a higher value indicating a greater probability that an exercise therapy is in the top rank (38). Funnel plots were generated to assess potential publication bias in NMA (Supplementary Appendix 7).

## 3 Results

### 3.1 Study selection

A total of 4,125 potentially eligible studies were identified, 4,087 articles of which were from database searches and 38 from reference list. After 2,268 duplicates were removed, 1,857 articles remained for screening. By screening the titles and abstracts, a further 1,801 articles were excluded. After reading the full text, 19 articles were deleted. Ultimately, 37 studies were included in this meta-analysis. Figure 1 presents the PRISMA flow diagram of the literature selection process.

### 3.2 Characteristics of the included studies

A total of 37 RCTs comprising 3483 participants were included in this study (Table 1). The trials were conducted in Asia (*n* = 20), Europe (*n* = 7), North America (*n* = 5), South America (*n* = 3), and Oceania (*n* = 2). Participants had a mean age ranging from 19 to 70.7 years. All individuals in the included studies were adults with stage 1 CKM syndrome, with characteristics of obesity (*n* = 13), overweight or obesity (*n* = 10), prediabetes (*n* = 11), prediabetes with overweight/ obesity (*n* = 3).

The methods for assessing VAT included bioelectrical impedance analysis (*n* = 9), computed tomography (*n* = 4), magnetic resonance imaging (*n* = 3), and dual-energy X-ray absorptiometry (*n* = 4). FBG was measured using the enzymatic method in some studies (*n* = 18), while other studies did not report the measurement method (*n* = 7). HbA1c was measured using immunochemical assay (*n* = 2), chromatography (*n* = 3), or methods not reported (*n* = 5) (Supplementary Appendix 8).

### 3.3 Results of ROB assessment and certainty of evidence

The design-by-treatment interaction model and node-splitting analysis indicated that direct and indirect bodies of evidence were consistent, with no clear violation of the transitivity assumption. In the risk-of-bias assessment, most of the studies (61.1%) were judged to have some concerns, while others were rated as low risk of bias (30.6%) or high risk of bias (8.3%) (Supplementary Appendix 9). CINeMA assessment showed that the certainty of evidence for VAT, FBG, and HbA1c was generally low to very low (Supplementary Appendix 10).

### 3.4 Network meta-analysis

With regard to the effects of different exercise modalities on VAT, FBG, and HbA1c in individuals with stage 1 CKM syndrome, a visual network plot was used to present the evidence base and its characteristics (68). In Figure 2, the diameter of each node is proportional to the sample size for each intervention, and the thickness of the lines represents the number of direct comparisons between pairs of interventions. Contributions of direct and indirect comparisons to the NMA and the number of studies on each direct comparison are provided in Supplementary Appendix 11.

**Figure 2:**
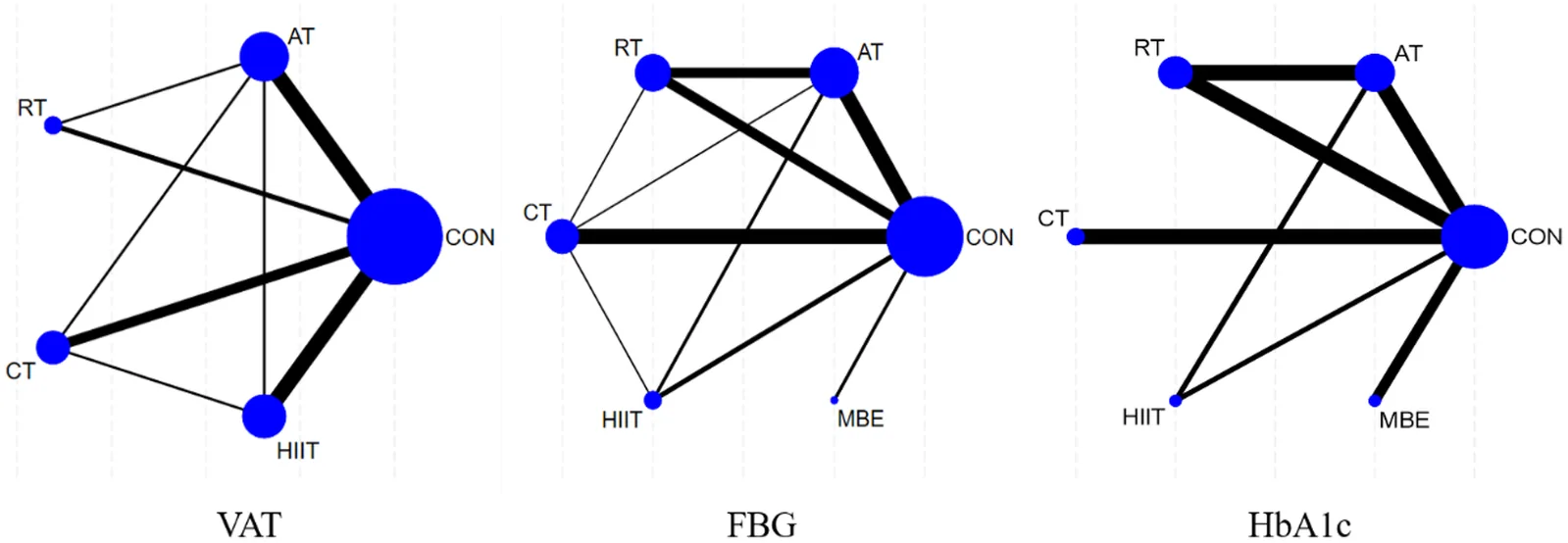
Network evidence plot of VAT, FBG, and HbA1c.

#### 3.4.1 Visceral adipose tissue

A total of 20 studies (*n* = 1,464) were included in this network meta-analysis. AT (SMD = −0.44, 95% CI: −0.62, −0.26), HIIT (SMD = −0.32, 95% CI: −0.56, −0.08), CT (SMD = −0.31, 95% CI: −0.51, −0.10), and RT (SMD = −0.27, 95% CI: −0.66, 0.12) showed improvements in VAT compared with the control group. Among them, statistically significant effects were observed in the AT, HIIT, and CT groups. The SUCRA probability ranking results showed that AT (SUCRA = 86.5%) had the highest probability of being the best exercise type for VAT, followed by HIIT (SUCRA = 57.8%), CT (SUCRA = 54.5%), and RT (SUCRA = 49%) (Table 2–3, Supplementary Appendix 12.1).

**Table 2:** Ranking of exercise intervention by order of effectiveness.

Treatment	SUCRA	PrBest (%)	Mean rank
VAT (20 studies, *N* = 1,464)			
AT	86.5	58.9	1.5
HIIT	57.8	14.9	2.7
CT	54.5	10	2.8
RT	49	16.2	3
CON	2.2	0	4.9
FBG (25 studies, *N* = 2,420)			
MBE	100	99.9	1
RT	64.8	0	2.8
AT	59.7	0	3
HIIT	41.8	0	3.9
CT	33.1	0	4.3
CON	0.6	0	6
HbA1c (10 studies, *N* = 1,370)			
MBE	78.4	44	2.1
CT	77.3	35	2.1
AT	67.2	16.1	2.6
RT	39.3	2.3	4
HIIT	31.4	2.7	4.4
CON	6.4	0	5.7

**Table 3:** Network meta-analysis matrix of VAT, FBG, HbA1c

**VAT**	AT	0.12 (−0.17, 0.41)		0.14 (−0.12, 0.39)	0.17 (−0.23, 0.57)	0.44 (0.26, 0.62)
	−0.12 (−0.41, 0.17)	HIIT		0.01 (−0.29, 0.32)	0.05 (−0.41, 0.50)	0.32 (0.08, 0.56)
	−0.14 (−0.39, 0.12)	−0.01 (−0.32, 0.29)		CT	0.03 (−0.40, 0.47)	0.31 (0.10, 0.51)
	−0.17 (−0.57, 0.23)	−0.05 (−0.50, 0.41)		−0.03 (−0.47, 0.40)	RT	0.27 (−0.12, 0.66)
	**−0.44 (−0.62, −0.26)**	**−0.32 (−0.56, −0.08)**		**−0.31 (−0.51, −0.10)**	−0.27 (−0.66, 0.12)	CON
**FBG**	MBE	0.80 (0.34,1.26)	0.83 (0.38, 1.27)	0.91 (0.43, 1.38)	0.95 (0.51, 1.39)	1.16 (0.75, 1.57)
	**−0.80 (−1.26, −0.34)**	RT	0.03 (−0.18, 0.24)	0.11 (−0.19, 0.41)	0.15 (−0.11, 0.41)	0.36 (0.14, 0.57)
	**−0.83 (−1.27, −0.38)**	−0.03 (−0.24, 0.18)	AT	0.08 (−0.18, 0.34)	0.12 (−0.10, 0.34)	0.33 (0.16, 0.50)
	**−0.91 (−1.38, −0.43)**	−0.11 (−0.41, 0.19)	−0.08 (−0.34, 0.18)	HIIT	0.04 (−0.23, 0.31)	0.25 (0.01, 0.49)
	**−0.95 (−1.39, −0.51)**	−0.15 (−0.41, 0.11)	−0.12 (−0.34, 0.10)	−0.04 (−0.31, 0.23)	CT	0.21 (0.05, 0.37)
	**−1.16 (−1.57, −0.75)**	**−0.36 (−0.57, −0.14)**	**−0.33 (−0.50, −0.16)**	**−0.25 (−0.49, −0.01)**	**−0.21 (−0.37, −0.05)**	CON
**HbA1c**	MBE	0.02 (−0.32, 0.35)	0.08 (−0.27, 0.43)	0.19 (−0.17, 0.55)	0.23 (−0.17, 0.63)	0.35 (0.07, 0.62)
	−0.02 (−0.35, 0.32)	CT	0.06 (−0.23, 0.36)	0.17 (−0.13, 0.48)	0.22 (−0.13, 0.57)	0.33 (0.14, 0.53)
	−0.08 (−0.43, 0.27)	−0.06 (−0.36, 0.23)	AT	0.11 (−0.11, 0.33)	0.16 (−0.14, 0.45)	0.27 (0.05, 0.49)
	−0.19 (−0.55, 0.17)	−0.17 (−0.48, 0.13)	−0.11 (−0.33, 0.11)	RT	0.05 (−0.29, 0.38)	0.16 (−0.07, 0.39)
	−0.23 (−0.63, 0.17)	−0.22 (−0.57, 0.13)	−0.16 (−0.45, 0.14)	−0.05 (−0.38, 0.29)	HIIT	0.12 (−0.18, 0.41)
	**−0.35 (−0.62, −0.07)**	**−0.33 (−0.53, −0.14)**	**−0.27 (−0.49, −0.05)**	−0.16 (−0.39, 0.07)	−0.12 (−0.41, 0.18)	CON

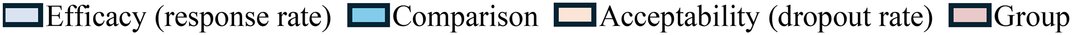

*Note:* Effects are expressed as the effect size [95% CI] between interventions. Bold indicates that the data are significant, Dark blue areas indicate the effect of the longitudinal versus the lateral intervention, Light blue areas indicate the effect of the lateral versus the longitudinal intervention, Grey areas represent the intervention category, and black areas represent the group. AT, aerobic training; RT, resistance training; CT, combined aerobic and resistance training; HIIT, high-intensity interval training; MBE, mind-body exercise; CON, control group.

#### 3.4.2 Fasting blood glucose

FBG was assessed in 25 studies (*n* = 2,420). Compared with the control group, MBE (MD = −1.16 mmol/L, 95% CI: −1.57, −0.75), RT (MD = −0.36 mmol/L, 95% CI: −0.57, −0.14), AT (MD = −0.33 mmol/L, 95% CI: −0.50, −0.16), HIIT (MD = −0.25 mmol/L, 95% CI: −0.49, −0.01), and CT (MD = −0.21 mmol/L, 95% CI: −0.37, −0.05) resulted in significant improvement in FBG. The SUCRA probability rankings indicated that MBE (SUCRA = 100%) had the highest probability of being the optimal exercise type for FBG, although the certainty of the evidence was limited. It was followed by RT (SUCRA = 64.8%), AT (SUCRA = 59.7%), HIIT (SUCRA = 41.8%), and CT (SUCRA = 33.1%) (Table 2–3, Supplementary Appendix 12.2).

#### 3.4.3 Glycated hemoglobin

A total of 10 studies (*n* = 1,370) were included in this network meta-analysis for HbA1c. MBE (MD = −0.35%, 95% CI: −0.62, −0.07), CT (MD = −0.33%, 95% CI: −0.53, −0.14), and AT (MD = −0.27%, 95% CI: −0.49, −0.05) significantly improved HbA1c compared with the control group. However, no significant difference was observed in the RT (MD = −0.16%, 95% CI: −0.39, 0.07) and HIIT (MD = −0.12%, 95% CI: −0.41, 0.18) compared with the control group. Meanwhile, the SUCRA probability sorting result indicated that MBE (SUCRA = 78.4%) had the highest probability of being best exercise type for HbA1c, followed by CT (SUCRA = 77.3%), AT (SUCRA = 67.2%), RT (SUCRA = 39.3%), and HIIT (SUCRA = 31.4%) (Table 2–3, Supplementary Appendix 12.3).

#### 3.4.4 Subgroup NMA of outcomes

We conducted subgroup analyses of VAT and FBG according to age and intervention duration. The inconsistency test results, SUCRA ranking, and NMA comparison matrices for the subgroup analyses are presented in Supplementary Appendices 13–15.

For the subgroup analysis of age, compared with the control group, only AT (SMD = −0.55, 95% CI: −0.72, −0.38) and CT (SMD = −0.28, 95% CI: −0.48, −0.08) significantly reduced VAT in individuals older than 35 years. In particular, HIIT showed the highest probability of being the optimal intervention for reducing VAT in participants aged ≤35 years (SUCRA = 86.7%), whereas AT had the highest probability of being the most efficacious exercise type for VAT reduction in participants aged >35 years (SUCRA = 96.5%).

For the intervention duration, compared with the control group, HIIT (SMD = −0.26, 95% CI: −0.49, −0.03) and AT (SMD = −0.25, 95% CI: −0.49, −0.02) significantly reduced VAT in interventions lasting ≤12 weeks, whereas AT (SMD = −0.64, 95% CI: −0.83, −0.44) and CT (SMD = −0.29, 95% CI: −0.50, −0.09) significantly reduced VAT in interventions lasting >12 weeks. The SUCRA probability ranking result indicated that HIIT (SUCRA = 67%) was most likely to be optimal for VAT reduction among interventions lasting ≤12 weeks, whereas AT was most likely to be the best among interventions lasting >12 weeks (SUCRA = 94.9%). In addition, compared with the control group, MBE (MD = −1.19 mmol/L, 95% CI: −1.67, −0.71) and AT (MD = −0.28 mmol/L, 95% CI: −0.50, −0.06) significantly improved FBG among interventions lasting ≤12 weeks, whereas MBE (MD = −1.04 mmol/L, 95% CI: −1.91, −0.17) and RT (MD = −0.53 mmol/L, 95% CI: −0.84, −0.21) significantly improved FBG in interventions lasting >12 weeks. Furthermore, MBE ranked highest for FBG improvement regardless of whether interventions lasted ≤12 weeks (SUCRA = 100%) or >12 weeks (SUCRA = 93.7%), but these results should be interpreted with caution.

### 3.5 Sensitivity analysis

We conducted sensitivity analyses, excluding studies with a high risk of bias, a possibly small proportion of non-target participants, or total exercise volume at the lower or upper extremes of the range. The SUCRA rankings for the different exercise types were generally consistent with the main results, supporting the robustness of the findings (Supplementary Appendix 16).

## 4 Discussion

This network meta-analysis suggests that exercise interventions can improve several cardiometabolic health outcomes in adults with stage 1 CKM syndrome. To our knowledge, this is the first network meta-analysis to compare the efficacy of different exercise modalities for reducing VAT and improving glucose parameters (FBG and HbA1c) in adults with stage 1 CKM syndrome.

The risk of obesity-related metabolic and cardiovascular complications is primarily driven by body fat distribution rather than total body adiposity. More adipose tissue deposition is associated with greater metabolic risk (69), and such deposition can be quantified using computed tomography (70). A recent study indicated that exercise interventions can improve glucose metabolism by reducing VAT without weight loss (71). Therefore, VAT has emerged as a critical target for regulating human metabolism. In fact, VAT mass is considered as a more accurate indicator of improvements in metabolic health than global adiposity or BMI (72, 73). However, evidence regarding the superiority of specific exercise types in reducing VAT remains unclear. Our findings showed that AT, HIIT, and CT contributed to a reduction in VAT compared with the control group, which was partially consistent with a previous study (74). Notably, CT was effective in reducing VAT in our analysis. The discrepancies between our results and those of previous studies may be attributed to the characteristics of the included CT interventions in this study, which featured sufficient exercise duration per session, a long intervention period, progressive exercise intensity, and a relatively high weekly exercise frequency (7, 55, 57). Mechanistically, the reduction of VAT induced by CT may be partially attributed to improvements in mitochondrial function (75, 76). Furthermore, in subgroup analyses stratified by age, our findings reported that HIIT appeared to be more beneficial for VAT reduction in individuals aged ≤35 years with stage 1 CKM syndrome, whereas AT and CT seemed to be more effective in those aged >35 years. These results provided some evidence that the effects of exercise on VAT reduction may differ by age in stage 1 CKM syndrome. In subgroup analyses stratified by intervention duration, interventions lasting more than 12 weeks appeared to be more effective in reducing VAT than those lasting 12 weeks or less for the same exercise type. This suggests that extending the intervention period may help enhance exercise-induced VAT loss.

Prediabetes is an abnormal metabolic state characterized by elevated glycemic levels that remain below the diagnostic threshold for type 2 diabetes (2, 77). Given the rising global prevalence of prediabetes, effective interventions are urgently required (78). An earlier study showed that glucose metabolism may be improved with adequate sleep, healthy diet, and regular physical activity among populations with prediabetes (79). In particular, evidence from RCTs suggests that AT (51, 61), RT (18, 58, 61), and MBE (52, 62) may enhance glycemic control in individuals with prediabetes. However, few studies compared the effects of different exercise types on glycemic levels through network meta-analysis. Partially consistent with earlier findings (80, 81), our study indicated that MBE showed the highest probability of being the best intervention for the reduction of FBG and HbA1c among all intervention types. Physiologically, the slow and rhythmic breathing movements of MBE may help improve parasympathetic nervous system function, regulate the hypothalamic–pituitary–adrenal axis (82, 83), and improve glycemic control (84). Moreover, an earlier study reported that FBG could be reduced through AT and RT in subjects with prediabetes (85), which supports our results. This improvement in FBG may be associated with increased insulin sensitivity (86) and the enhanced ability of glucose transporters to take up glucose (87). In addition, our results demonstrated that CT also may improve HbA1c levels in individuals with stage 1 CKM syndrome, in partial agreement with a previous study (88). The result may be attributable to the characteristics of the CT protocol, including a frequency of 2–3 sessions per week, a duration of 4–6 months, and moderate-to-vigorous intensity exercise (7, 54). Mechanistically, this combined exercise may demonstrate greater efficacy in improving insulin sensitivity compared with aerobic or resistance training alone (89).

## 5 Limitations

This study has several limitations that should be considered when interpreting our findings. First, the literature search was limited to Chinese and English publications, excluding other languages, trial registries, CENTRAL, and gray literature. This may have resulted in the omission of relevant literature and affected this meta-analysis. Second, the study primarily assessed the independent effects of exercise interventions on VAT, FBG, and HbA1c. However, a correlation analysis between the reduction in VAT and the improvement in glucose metabolism was not performed. Finally, due to the limited number of included studies, subgroup analyses of HbA1c could not be conducted. Consequently, we were unable to assess heterogeneity and examine the differential effects of exercise across subgroups defined by age and intervention duration. Therefore, the results should be cautiously interpreted. Given the limitations of this study, analyses with larger sample sizes should be performed. Moreover, the relationship between exercise-induced reduction in visceral fat and improvements in glucose metabolism should be explored in the future.

## 6 Conclusion

The present NMA provides evidence that exercise interventions confer beneficial effects for individuals with stage 1 CKM syndrome, potentially helping improve metabolic risk factors relevant to early CKM health. In this study, aerobic training was found to be most effective in reducing the accumulation of VAT, while mind–body exercise may have offered more benefits in lowering FBG and HbA1c.

## Data Availability

The original contributions presented in the study are included in the article/Supplementary Material, further inquiries can be directed to the corresponding authors.

## References

[1] Ndumele CE Neeland IJ Tuttle KR Chow SL Mathew RO Khan SS. A synopsis of the evidence for the science and clinical management of cardiovascular-kidney-metabolic (CKM) syndrome: a scientific statement from the American Heart Association. Circulation. (2023) 148(20):1636–64. 10.1161/CIR.0000000000001186 37807920

[2] Ndumele CE Rangaswami J Chow SL Neeland IJ Tuttle KR Khan SS. Cardiovascular-kidney-metabolic health: a presidential advisory from the American Heart Association. Circulation. (2023) 148(20):1606–35. 10.1161/CIR.0000000000001184 37807924

[3] Kim J-E Joo J Kuku KO Downie C Hashemian M Powell-Wiley TM. Prevalence, disparities, and mortality of cardiovascular-kidney-metabolic syndrome in US adults, 2011–2018. Am J Med. (2025) 138(6):970–9.e7. 10.1016/j.amjmed.2025.01.031 39909293 PMC12092198

[4] Zhu R Wang R He J Wang L Chen H Niu X. Prevalence of cardiovascular-kidney-metabolic syndrome stages by social determinants of health. JAMA Netw Open. (2024) 7(11):e2445309. 10.1001/jamanetworkopen.2024.45309 39556396 PMC11574692

[5] Huang Q Li Y Zhou X Zou X Ji L. Prevalence of the cardiovascular-kidney-metabolic syndrome in 3 Chinese cohorts. JACC Asia. (2025) 5(1):116–8. 10.1016/j.jacasi.2024.10.016 39886203 PMC11775807

[6] Jadhav RA Maiya GA Shivashankara KN Umakanth S. Measurement of visceral fat for early prediction of prediabetes—cross-sectional study from southern India. J Taibah Univ Med Sci. (2022) 17(6):983–90. 10.1016/j.jtumed.2022.05.006 36212585 PMC9519599

[7] Jadhav RA Maiya GA Umakanth S Shivashankara KN. Effect of a customized physical activity promotion program on visceral fat and glycemic parameters in individuals with prediabetes: a randomized controlled trial. J Phys Act Health. (2024) 21(2):164–70. 10.1123/jpah.2023-0486 38016451

[8] Park HS Lee K. Greater beneficial effects of visceral fat reduction compared with subcutaneous fat reduction on parameters of the metabolic syndrome: a study of weight reduction programmes in subjects with visceral and subcutaneous obesity. Diabet Med. (2005) 22(3):266–72. 10.1111/j.1464-5491.2004.01395.x 15717873

[9] Lingvay I Cohen RV Roux CWL Sumithran P. Obesity in adults. Lancet. (2024) 404(10456):972–87. 10.1016/S0140-6736(24)01210-8 39159652

[10] Hirani K Sundheim B Blaschke M Lemos JRN Mittal R. A global call to action: strengthening strategies to combat dysglycemia and improve public health outcomes. Front Public Health. (2025) 13:1597128. 10.3389/fpubh.2025.1597128 40520321 PMC12162950

[11] Courcoulas AP Daigle CR Arterburn DE. Long term outcomes of metabolic/bariatric surgery in adults. Br Med J. (2023) 383:e071027. 10.1136/bmj-2022-071027 38110235

[12] Færch K Blond MB Bruhn L Amadid H Vistisen D Clemmensen KKB. The effects of dapagliflozin, metformin or exercise on glycaemic variability in overweight or obese individuals with prediabetes (the pre-d trial): a multi-arm, randomised, controlled trial. Diabetologia. (2021) 64(1):42–55. 10.1007/s00125-020-05306-1 33064182

[13] Papadakis Z. Exercise in CKM syndrome progression: a stage-specific approach to cardiovascular, metabolic, and renal health. Cardiovasc Diabetol. (2025) 24(1):462. 10.1186/s12933-025-03029-4 41421989 PMC12739863

[14] Thyfault JP Bergouignan A. Exercise and metabolic health: beyond skeletal muscle. Diabetologia. (2020) 63(8):1464–74. 10.1007/s00125-020-05177-6 32529412 PMC7377236

[15] Wedell-Neergaard A-S Lang Lehrskov L Christensen RH Legaard GE Dorph E Larsen MK. Exercise-induced changes in visceral adipose tissue mass are regulated by il-6 signaling: a randomized controlled trial. Cell Metab. (2019) 29(4):844–55.e3. 10.1016/j.cmet.2018.12.007 30595477

[16] Borror A Zieff G Battaglini C Stoner L. The effects of postprandial exercise on glucose control in individuals with type 2 diabetes: a systematic review. Sports Med. (2018) 48(6):1479–91. 10.1007/s40279-018-0864-x 29396781

[17] Chen X Wu L Zheng Y Ni X Zhuang X Chen L. Effective of high-intensity interval training and moderate-intensity continuous training on body composition, glycolipid metabolism, and cardiopulmonary function in patients with pre-diabetes: a randomized controlled trial. Front Endocrinol (Lausanne). (2025) 16:1614149. 10.3389/fendo.2025.1614149 40801034 PMC12339354

[18] Luo X Wang Z Li B Zhang X Li X. Effect of resistance vs. aerobic exercise in pre-diabetes: an rct. Trials. (2023) 24(1):110. 10.1186/s13063-023-07116-3 36788568 PMC9930288

[19] Morey MC Pieper CF Edelman DE Yancy WS Green JB Lum H. Enhanced fitness: a randomized controlled trial of the effects of home-based physical activity counseling on glycemic control in older adults with prediabetes mellitus. J Am Geriatr Soc. (2012) 60(9):1655–62. 10.1111/j.1532-5415.2012.04119.x 22985140 PMC3448120

[20] Ballin M Lundberg E Sörlén N Nordström P Hult A Nordström A. Effects of interval training on visceral adipose tissue in centrally obese 70-year-old individuals: a randomized controlled trial. J Am Geriatr Soc. (2019) 67(8):1625–31. 10.1111/jgs.15919 31012497

[21] Nunes PRP Martins FM Souza AP Carneiro MAS Orsatti CL Michelin MA. Effect of high-intensity interval training on body composition and inflammatory markers in obese postmenopausal women: a randomized controlled trial. Menopause. (2019) 26(3):256–64. 10.1097/GME.0000000000001207 30277921

[22] Haganes KL Silva CP Eyjólfsdóttir SK Steen S Grindberg M Lydersen S. Time-restricted eating and exercise training improve hba1c and body composition in women with overweight/obesity: a randomized controlled trial. Cell Metab. (2022) 34(10):1457–71.e4. 10.1016/j.cmet.2022.09.003 36198292

[23] Chow BC Li S Zhu X Jiao J Quach B Baker JS. Effects of descending or ascending stair exercise on body composition, insulin sensitivity, and inflammatory markers in young Chinese women with obesity: a randomized controlled trial. J Sports Sci. (2021) 39(5):496–502. 10.1080/02640414.2020.1829362 33012244

[24] Tonin FS Rotta I Mendes AM Pontarolo R. Network meta-analysis: a technique to gather evidence from direct and indirect comparisons. Pharm Pract (Granada). (2017) 15(1):943. 10.18549/PharmPract.2017.01.943 28503228 PMC5386629

[25] Hutton B Salanti G Caldwell DM Chaimani A Schmid CH Cameron C. The PRISMA extension statement for reporting of systematic reviews incorporating network meta-analyses of health care interventions: checklist and explanations. Ann Intern Med. (2015) 162(11):777–84. 10.7326/M14-2385 26030634

[26] Ndumele CE Rodriguez F Dixon DL Khan SS Mukherjee D Bajaj M. 2026 AHA/ACC/ADA/ASN guideline for the prevention, detection, evaluation, and management of cardiovascular-kidney-metabolic syndrome: a report of the American College of Cardiology/American Heart Association joint committee on clinical practice guidelines. Circulation. (2026) 154(4):50–158. 10.1161/cir.0000000000001453 42263157

[27] Sterne JAC Savović J Page MJ Elbers RG Blencowe NS Boutron I. RoB 2: a revised tool for assessing risk of bias in randomised trials. Br Med J. (2019) 366:l4898. 10.1136/bmj.l4898 31462531

[28] Nikolakopoulou A Higgins JPT Papakonstantinou T Chaimani A Del Giovane C Egger M. CINeMA: an approach for assessing confidence in the results of a network meta-analysis. PLoS Med. (2020) 17(4):e1003082. 10.1371/journal.pmed.1003082 32243458 PMC7122720

[29] Salanti G Del Giovane C Chaimani A Caldwell DM Higgins JP. Evaluating the quality of evidence from a network meta-analysis. PLoS One. (2014) 9(7):e99682. 10.1371/journal.pone.0099682 24992266 PMC4084629

[30] Higgins JPT Li T Deeks JJ. Choosing effect measures and computing estimates of effect. In: Higgins JPT and Green S, editors. Cochrane Handbook for Systematic Reviews of Interventions. London: Cochrane Collaboration (2019). p. 143–76. 10.1002/9781119536604.ch6

[31] Shi C Westby M Norman G Dumville JC Cullum N. Node-making processes in network meta-analysis of nonpharmacological interventions should be well planned and reported. J Clin Epidemiol. (2018) 101:124–5. 10.1016/j.jclinepi.2018.04.009 29678507

[32] Jackson D Riley R White IR. Multivariate meta-analysis: potential and promise. Stat Med. (2011) 30(20):2481–98. 10.1002/sim.4172 21268052 PMC3470931

[33] Higgins JP Thompson SG Deeks JJ Altman DG. Measuring inconsistency in meta-analyses. Br Med J. (2003) 327(7414):557–60. 10.1136/bmj.327.7414.557 PMC192859 12958120

[34] Salanti G. Indirect and mixed-treatment comparison, network, or multiple-treatments meta-analysis: many names, many benefits, many concerns for the next generation evidence synthesis tool. Res Synth Methods. (2012) 3(2):80–97. 10.1002/jrsm.1037 26062083

[35] White IR Barrett JK Jackson D Higgins JP. Consistency and inconsistency in network meta-analysis: model estimation using multivariate meta-regression. Res Synth Methods. (2012) 3(2):111–25. 10.1002/jrsm.1045 26062085 PMC4433771

[36] Shim S Yoon BH Shin IS Bae JM. Network meta-analysis: application and practice using Stata. Epidemiol Health. (2017) 39:e2017047. 10.4178/epih.e2017047 29092392 PMC5733388

[37] Salanti G Ades AE Ioannidis JP. Graphical methods and numerical summaries for presenting results from multiple-treatment meta-analysis: an overview and tutorial. J Clin Epidemiol. (2011) 64(2):163–71. 10.1016/j.jclinepi.2010.03.016 20688472

[38] Mbuagbaw L Rochwerg B Jaeschke R Heels-Andsell D Alhazzani W Thabane L. Approaches to interpreting and choosing the best treatments in network meta-analyses. Syst Rev. (2017) 6(1):79. 10.1186/s13643-017-0473-z 28403893 PMC5389085

[39] Guo Z Tan S. Effect of 12-week advanced weight loss exercise on cardiopulmonary function in obese young men. J Tianjin Univ Sport. (2021) 36(4):490–6. 10.13297/j.cnki.issn1005-0000.2021.04.018

[40] Lin J Zhao HQ Huang XA Liu X Zhang R. Effect of high intensity interval training on the body composition, blood lipid and fasting insulin level of obese female. College Stud Chin Gen Pract. (2016) 19(18):2139–44. 10.3969/j.issn.1007-9572.2016.18.006

[41] Dai W. Research on the effect of loss weight with high intensity intermittent swimming on obese young women. J Guangzhou Sport Univ. (2014) 34(6):99–102. 10.13830/j.cnki.cn44-1129/g8.2014.06.026

[42] Qi YG Wang J Xu DQ. Comparison of the effects of aerobic-resistance and aerobic training on body weight control in female university students with obesity. J Tianjin Univ Sport. (2020) 35(5):541–4. 10.13297/j.cnki.issn1005-0000.2020.05.008.43

[43] Tong TK Zhang H Shi H Liu Y Ai J Nie J. Comparing time efficiency of sprint vs. High-intensity interval training in reducing abdominal visceral fat in obese young women: a randomized, controlled trial. Front Physiol. (2018) 9:1048. 10.3389/fphys.2018.01048 30123136 PMC6085472

[44] Chiang TL Chen C Hsu CH Lin YC Wu HJ. Is the goal of 12,000 steps per day sufficient for improving body composition and metabolic syndrome? The necessity of combining exercise intensity: a randomized controlled trial. BMC Public Health. (2019) 19(1):1215. 10.1186/s12889-019-7554-y 31481039 PMC6724241

[45] Ross R Janssen I Dawson J Kungl A-M Kuk JL Wong SL. Exercise-induced reduction in obesity and insulin resistance in women: a randomized controlled trial. Obes Res. (2004) 12(5):789–98. 10.1038/oby.2004.95 15166299

[46] Friedenreich CM Woolcott CG McTiernan A Terry T Brant R Ballard-Barbash R. Adiposity changes after a 1-year aerobic exercise intervention among postmenopausal women: a randomized controlled trial. Int J Obes. (2011) 35(3):427–35. 10.1038/ijo.2010.147 PMC3061001 20820172

[47] Keating SE Hackett DA Parker HM Way KL O'Connor HT Sainsbury A. Effect of resistance training on liver fat and visceral adiposity in adults with obesity: a randomized controlled trial. Hepatol Res. (2017) 47(7):622–31. 10.1111/hepr.12781 27480242

[48] Han D Wang XY. Application of exercise intervention program based on smart phone APP in prediabetic patients. Chin Nurs Res. (2022) 36(1):128–32. 10.12102/j.issn.1009-6493.2022.01.025

[49] Wang ZZ Wang Y. Influence of aerobic exercise on the insulin sensitivity of pre-diabetes group. J Chengdu Sport Univ. (2013) 39(9):1–8. 10.15942/j.jcsu.2013.09.003

[50] Wu Y. The effects of different exercise modes on insulin sensitivity and physical performance in elderly individuals with impaired glucose regulation. J Southwest Univ. (Natural Science Edition) (2018) 43(8):83–91. 10.13718/j.cnki.xsxb.2018.08.015

[51] Yan J Dai X Feng J Yuan X Li J Yang L. Effect of 12-month resistance training on changes in abdominal adipose tissue and metabolic variables in patients with prediabetes: a randomized controlled trial. J Diabetes Res. (2019) 2019:8469739. 10.1155/2019/8469739 31737686 PMC6815994

[52] Karaman A Angın E. Effects of clinical Pilates exercises on glycemic control, blood lipids, and physical fitness parameters in women with prediabetes: a randomized controlled trial. Physiother Theory Pract. (2026) 42(4):528–41. 10.1080/09593985.2025.2591158 41293814

[53] Herzig KH Ahola R Leppäluoto J Jokelainen J Jämsä T Keinänen-Kiukaanniemi S. Light physical activity determined by a motion sensor decreases insulin resistance, improves lipid homeostasis and reduces visceral fat in high-risk subjects: Prediabex study RCT. Int J Obes (Lond). (2014) 38(8):1089–96. 10.1038/ijo.2013.224 24285336 PMC4125749

[54] Hafeez S Rehman SSU Riaz S Hafeez I Hafeez Z Mumtaz H. Impact of exercise manual program on biochemical markers in sedentary prediabetic patients: a randomized controlled trial. Medicina (Kaunas). (2025) 61(2):190. 10.3390/medicina61020190 40005307 PMC11857685

[55] Irwin ML Yasui Y Ulrich CM Bowen D Rudolph RE Schwartz RS. Effect of exercise on total and intra-abdominal body fat in postmenopausal women: a randomized controlled trial. JAMA. (2003) 289(3):323–30. 10.1001/jama.289.3.323 12525233

[56] Chen X Zhao S Hsue C Dai X Liu L Miller JD. Effects of aerobic training and resistance training in reducing cardiovascular disease risk for patients with prediabetes: a multi-center randomized controlled trial. Prim Care Diabetes. (2021) 15(6):1063–70. 10.1016/j.pcd.2021.08.013 34649825

[57] Bouchonville M Armamento-Villareal R Shah K Napoli N Sinacore DR Qualls C. Weight loss, exercise or both and cardiometabolic risk factors in obese older adults: results of a randomized controlled trial. Int J Obes (Lond). (2014) 38(3):423–31. 10.1038/ijo.2013.122 23823329 PMC3835728

[58] Tomeleri CM Ribeiro AS Souza MF Schiavoni D Schoenfeld BJ Venturini D. Resistance training improves inflammatory level, lipid and glycemic profiles in obese older women: a randomized controlled trial. Exp Gerontol. (2016) 84:80–7. 10.1016/j.exger.2016.09.005 27616162

[59] Streb AR Dos Santos Leonel L Delevatti RS Cavaglieri CR Del Duca GF. Effects of non-periodized and linear periodized combined exercise training on insulin resistance indicators in adults with obesity: a randomized controlled trial. Sports Med Open. (2021) 7(1):69. 10.1186/s40798-021-00359-x 34568974 PMC8473518

[60] Ratajczak M Krzywicka M Szulińska M Musiałowska D Kusy K Karolkiewicz J. Effects of 12-week combined strength and endurance circuit training program on insulin sensitivity and retinol-binding protein 4 in women with insulin-resistance and overweight or mild obesity: a randomized controlled trial. Diabetes Metab Syndr Obes. (2024) 17:93–106. 10.2147/DMSO.S432954 38204866 PMC10778163

[61] Yuan X Dai X Liu L Hsue C Miller JD Fang Z. Comparing the effects of 6 months aerobic exercise and resistance training on metabolic control and *β*-cell function in Chinese patients with prediabetes: a multicenter randomized controlled trial. J Diabetes. (2020) 12(1):25–37. 10.1111/1753-0407.12955 31141300

[62] Sawangwong P Tungsukruthai S Nootim P Sriyakul K Phetkate P Pawa KK. The effects of 12-week traditional Thai exercise (Ruesi Dadton) on glycemic control and inflammatory markers in prediabetes: a randomized controlled trial. Life (Basel). (2023) 13(11):2166. 10.3390/life13112166 38004306 PMC10672481

[63] Siu PM Leung CK Bernal JDK Yu AP Recchia F Tam BT. Once and thrice weekly interval training in adults with central obesity: a randomized controlled trial. Nat Commun. (2026) 17(1):1410. 10.1038/s41467-025-68149-7 41519797 PMC12881463

[64] Shahiddoust F Monazzami AA. Exercise-induced changes in insulin sensitivity, atherogenic index of plasma, and CTRP1/CTRP3 levels: the role of combined and high-intensity interval training in overweight and obese women. BMC Sports Sci Med Rehabil. (2025) 17(1):73. 10.1186/s13102-025-01123-4 40200367 PMC11977882

[65] Ho SS Dhaliwal SS Hills AP Pal S. The effect of 12 weeks of aerobic, resistance or combination exercise training on cardiovascular risk factors in the overweight and obese in a randomized trial. BMC Public Health. (2012) 12:704. 10.1186/1471-2458-12-704 23006411 PMC3487794

[66] De Strijcker D Lapauw B Ouwens DM Van de Velde D Hansen D Petrovic M. High intensity interval training is associated with greater impact on physical fitness, insulin sensitivity and muscle mitochondrial content in males with overweight/obesity, as opposed to continuous endurance training: a randomized controlled trial. J Musculoskelet Neuronal Interact. (2018) 18(2):215–26. 29855444 PMC6016496

[67] Tan A Dunseath G Thomas RL Prior SL Bracken RM Churm R. Effect of home-based exercise with or without a Mediterranean-style diet on adiposity markers in postmenopausal women: a randomized-control trial. Physiol Rep. (2025) 13(3):e70239. 10.14814/phy2.70239 39921234 PMC11805804

[68] Chaimani A Higgins JP Mavridis D Spyridonos P Salanti G. Graphical tools for network meta-analysis in Stata. PLoS One. (2013) 8(10):e76654. 10.1371/journal.pone.0076654 24098547 PMC3789683

[69] Jensen MD. Role of body fat distribution and the metabolic complications of obesity. J Clin Endocrinol Metab. (2008) 93(11 Suppl 1):S57–63. 10.1210/jc.2008-1585 18987271 PMC2585758

[70] Graffy PM Pickhardt PJ. Quantification of hepatic and visceral fat by CT and MR imaging: relevance to the obesity epidemic, metabolic syndrome and NAFLD. Br J Radiol. (2016) 89(1062):20151024. 10.1259/bjr.20151024 26876880 PMC5258166

[71] Sandforth A Arreola EV Hanson RL Wewer Albrechtsen NJ Holst JJ Ahrends R. Prevention of type 2 diabetes through prediabetes remission without weight loss. Nat Med. (2025) 31(10):3330–40. 10.1038/s41591-025-03944-9 41023486 PMC12532634

[72] Kim SK Kim HJ Hur KY Choi SH Ahn CW Lim SK. Visceral fat thickness measured by ultrasonography can estimate not only visceral obesity but also risks of cardiovascular and metabolic diseases. Am J Clin Nutr. (2004) 79(4):593–9. 10.1093/ajcn/79.4.593 15051602

[73] Kang SH Cho KH Park JW Yoon KW Do JY. Association of visceral fat area with chronic kidney disease and metabolic syndrome risk in the general population: analysis using multi-frequency bioimpedance. Kidney Blood Press Res. (2015) 40(3):223–30. 10.1159/000368498 25966816

[74] Chang YH Yang HY Shun SC. Effect of exercise intervention dosage on reducing visceral adipose tissue: a systematic review and network meta-analysis of randomized controlled trials. Int J Obes (Lond). (2021) 45(5):982–97. 10.1038/s41366-021-00767-9 33558643

[75] Waters DL Aguirre L Gurney B Sinacore DR Fowler K Gregori G. Effect of aerobic or resistance exercise, or both, on intermuscular and visceral fat and physical and metabolic function in older adults with obesity while dieting. J Gerontol A Biol Sci Med Sci. (2022) 77(1):131–9. 10.1093/gerona/glab111 33839788 PMC8751785

[76] Huffman KM Koves TR Hubal MJ Abouassi H Beri N Bateman LA. Metabolite signatures of exercise training in human skeletal muscle relate to mitochondrial remodelling and cardiometabolic fitness. Diabetologia. (2014) 57(11):2282–95. 10.1007/s00125-014-3343-4 25091629 PMC4182127

[77] World Health Organization. Definition and diagnosis of diabetes mellitus and intermediate hyperglycaemia: report of a WHO/IDF consultation (2006). Available online at: https://www.who.int/publications/i/item/definition-and-diagnosis-of-diabetes-mellitus-and-intermediate-hyperglycaemia (Accessed April 10, 2026).

[78] Saeedi P Petersohn I Salpea P Malanda B Karuranga S Unwin N. Global and regional diabetes prevalence estimates for 2019 and projections for 2030 and 2045: results from the international diabetes federation diabetes atlas, 9(th) edition. Diabetes Res Clin Pract. (2019) 157:107843. 10.1016/j.diabres.2019.107843 31518657

[79] Rosenfeld RM Grega ML Karlsen MC Abu Dabrh AM Aurora RN Bonnet JP. Lifestyle interventions for treatment and remission of type 2 diabetes and prediabetes in adults: a clinical practice guideline from the American College of Lifestyle Medicine. Am J Lifestyle Med. (2025) 19(2 Suppl):10s–131. 10.1177/15598276251325488 40546761 PMC12178993

[80] Yang Y Chen R Wang X Yan Y Huang T Wang C. Mind-body interventions for prediabetes management: traditional Chinese exercise and its dual effects on metabolic control and psychological well-being-a systematic review and meta-analysis. J Diabetes Res. (2025) 2025:8249301. 10.1155/jdr/8249301 41255514 PMC12623101

[81] Luo J Tao Y Xu Y Yang L Zhang H Chen Y. Global comparison of the effect of non-pharmacological interventions on glycemic control in patients with type 2 diabetes: a network meta-analysis from 107 randomized controlled trials. J Diabetes Complications. (2023) 37(7):108518. 10.1016/j.jdiacomp.2023.108518 37267719

[82] Inbaraj G Bajaj S Misra P Kandimalla N Thapa A Ghosal A. Yoga in obesity management: reducing cardiovascular risk and enhancing well-being- a review of the current literature. Curr Probl Cardiol. (2025) 50(6):103036. 10.1016/j.cpcardiol.2025.103036 40132783

[83] Kiecolt-Glaser JK Christian L Preston H Houts CR Malarkey WB Emery CF. Stress, inflammation, and yoga practice. Psychosom Med. (2010) 72(2):113–21. 10.1097/PSY.0b013e3181cb9377 20064902 PMC2820143

[84] Rajput R Yadav K Rajput M Yadav J Saini N. Effect of yoga on glucose control and quality of life in patients of prediabetes. Metab Syndr Relat Disord. (2021) 19(8):417–21. 10.1089/met.2020.0040 34037489

[85] Liu L Ma X Xu H Ruan S Yuan X. Comparing the effects of 12 months aerobic exercise and resistance training on glucose metabolism among prediabetes phenotype: a explorative randomized controlled trial. Prim Care Diabetes. (2021) 15(2):340–6. 10.1016/j.pcd.2020.11.003 33309489

[86] Bird SR Hawley JA. Update on the effects of physical activity on insulin sensitivity in humans. BMJ Open Sport Exerc Med. (2017) 2(1):e000143. 10.1136/bmjsem-2016-000143 28879026 PMC5569266

[87] Yang J. Enhanced skeletal muscle for effective glucose homeostasis. Prog Mol Biol Transl Sci. (2014) 121:133–63. 10.1016/B978-0-12-800101-1.00005-3 24373237

[88] Yan R Li Y Jia S He J Lin G Huang W. Optimal exercise dose for glycemic control in prediabetes across different exercise types. iScience. (2025) 28(12):113980. 10.1016/j.isci.2025.113980 41377666 PMC12689185

[89] Inoue DS De Mello MT Foschini D Lira FS De Piano Ganen A Da Silveira Campos RM. Linear and undulating periodized strength plus aerobic training promote similar benefits and lead to improvement of insulin resistance on obese adolescents. J Diabetes Complications. (2015) 29(2):258–64. 10.1016/j.jdiacomp.2014.11.002 25441178

