# Supplementary material for: Effects of different exercise types on visceral adipose tissue and glycemic parameters in adults with stage 1 cardiovascular-kidney-metabolic syndrome: a systematic review and network meta-analysis of randomized controlled trials
Source: Front Cardiovasc Med. 2026 Sep 29;13:1873140. doi: 10.3389/fcvm.2026.1873140 (PMC13659888; doi:10.3389/fcvm.2026.1873140)
Supplement: Supplementary file 1 [file Datasheet1.pdf]

Effects of different exercise types on visceral adipose tissue and plasma glucose in adults with stage 1 of Cardiovascular-Kidney-Metabolic (CKM) syndrome: a systematic review and network meta-analysis of randomized controlled trials

Xinda WEI[1], Hanyu ZHAO[1], Shuang YUE[1], Tao REN[1], Yuhang YANG[1], Jiazhen WANG[1], Ruihua CAO[1], Ji FANG[1], Fan SU[1,2,*], Fei ZHONG[1,2,*]

[1]College of Physical Education, China Three Gorges University, Yichang, China;
[2] Institute of Sports Rehabilitation, China Three Gorges University, Yichang, China

[*]Corresponding author

Fan Su, College of Physical Education, China Three Gorges University, 8 Daxue Road, Yichang 443000, China.

Email: edu0114@126.com

Fei Zhong, College of Physical Education, China Three Gorges University, 8 Daxue Road, Yichang 443000, China.

Email: zf358235743@zju.edu.cn

Content

Appendix 1 Search strategy of Pubmed, Web of Science, Embase, CNKI, WANFANG Database, VIP Database.. 3
Appendix 2 Definitions of exercise interventions 6
Appendix 3 Transformation formulas by exercise type.. 7
Appendix 4 Forest plot comparing exercise versus control for VAT, FBG, and HbA1c. 8
Appendix 5 Prediction interval forest plots for VAT, FBG and HbA1c 9
Appendix 6 Inconsistency Models, Loop-Specific Heterogeneity, and Node-Splitting Analyses for VAT, FBG and HbA1c. 12
Appendix 7 The funnel plot graphics of VAT, FBG and HbA1c in NMA............................. 15
Appendix 8 Measurement methods for different outcome variables..................................... 17
Appendix 9 Risk assessment of Bias in the included Studies 18
Appendix 10 Results of the CINeMA assessment for VAT, FBG and HbA1c 20
Appendix 11 Contributions of direct and indirect comparisons to NMA and the number of studies per comparison for VAT, FBG and HbA1c... 23
Appendix 12 SUCRA ranking for VAT, FBG and HbA1c... 26
Appendix 13 Inconsistency model for subgroups of VAT and FBG...................................... 29
Appendix 14 Ranking in the subgroup of VAT and FBG 30
Appendix 15 Network meta-analysis matrix for subgroup analysis of VAT and FBG........... 31
Appendix 16 Sensitivity analysis for VAT, FBG, and HbA1c 34

Appendix 1 Search strategy of Pubmed, Web of Science, Embase, CNKI, WANFANG Database, VIP Database.

Appendix 1.1 Search strategy in PubMed (*N* = 1118)

Search strategy	Results
Search: (((prediabetes[Title/Abstract] OR prediabetic[Title/Abstract] OR prediabetic state[Title/Abstract] OR impaired fasting glucose[Title/Abstract] OR impaired glucose tolerance[Title/Abstract] OR obesity[Title/Abstract] OR obese[Title/Abstract] OR overweight[Title/Abstract] OR visceral adiposity[Title/Abstract] OR abdominal obesity[Title/Abstract] OR central obesity[Title/Abstract] OR waist circumference[Title/Abstract] OR metabolic syndrome[Title/Abstract]) AND (exercise OR physical activity OR physically active OR training OR aerobic exercise OR resistance training OR strength training OR high-intensity interval training OR walking OR jogging OR swimming OR cycling OR mind-body exercise OR tai chi OR qigong OR baduanjin OR wuqinxi OR yijinjing OR yoga OR traditional chinese exercise OR martial arts[MeSH Terms])) AND (Abdominal Fat OR Intra-Abdominal Fat OR visceral fat OR visceral adipose tissue OR visceral adiposity OR intra-abdominal fat OR abdominal fat OR Blood Glucose OR Glycemic Control OR fasting glucose OR fasting blood glucose OR FPG OR impaired fasting glucose OR blood glucose OR Glycated Hemoglobin A OR HbA1c OR hemoglobin A1c OR glycated hemoglobin[MeSH Terms])) AND (Randomized Controlled Trial[Title/Abstract] OR Controlled Clinical Trial[Title/Abstract]) Sort by: Publication Date	1118

Appendix 1.2 Search strategy in web of science (*N* = 982)

Search strategy	Results
**Topic:** prediabetes OR prediabetic OR prediabetic state OR impaired fasting glucose OR impaired glucose tolerance OR obesity OR obese OR overweight OR visceral adiposity OR abdominal obesity OR central obesity OR waist circumference OR metabolic syndrome **and Topic:** exercise OR physical activity OR physically active OR training OR aerobic exercise OR resistance training OR strength training OR high-intensity interval training OR walking OR jogging OR swimming OR cycling OR mind-body exercise OR tai chi OR qigong OR baduanjin OR wuqinxi OR yijinjing OR yoga OR traditional chinese exercise OR martial arts **and Topic:** Abdominal Fat OR Intra-Abdominal Fat OR visceral fat OR visceral adipose tissue OR visceral adiposity OR intra-abdominal fat OR abdominal fat OR Blood Glucose OR Glycemic Control OR fasting glucose OR fasting blood glucose OR FPG OR impaired fasting glucose OR blood glucose OR Glycated Hemoglobin A OR HbA1c OR hemoglobin A1c OR glycated hemoglobin **and Title:** Randomized Controlled Trial or Controlled Clinical Trial.	982

Appendix 1.3 Search strategy in Embase (N = 936)

Search strategy	Results
(prediabetes:ti,ab,kw OR prediabetic:ti,ab,kw OR 'prediabetic state':ti,ab,kw OR 'impaired fasting glucose':ti,ab,kw OR 'impaired glucose tolerance':ti,ab,kw OR obesity:ti,ab,kw OR obese:ti,ab,kw OR overweight:ti,ab,kw OR 'visceral adiposity':ti,ab,kw OR 'abdominal obesity':ti,ab,kw OR 'central obesity':ti,ab,kw OR 'waist circumference':ti,ab,kw OR 'metabolic syndrome':ti,ab,kw) AND (exercise:ab,ti OR 'physical activity':ab,ti OR 'physically active':ab,ti OR training:ab,ti OR 'aerobic exercise':ab,ti OR 'resistance training':ab,ti OR 'strength training':ab,ti OR 'high-intensity interval training':ab,ti OR walking:ab,ti OR jogging:ab,ti OR swimming:ab,ti OR cycling:ab,ti OR 'mind-body exercise':ab,ti OR 'tai chi':ab,ti OR qigong:ab,ti OR baduanjin:ab,ti OR wuqinxi:ab,ti OR yijinjing:ab,ti OR yoga:ab,ti OR 'traditional chinese exercise':ab,ti OR 'martial arts':ab,ti) AND ('visceral fat':ab,ti OR 'visceral adipose tissue':ab,ti OR 'visceral adiposity':ab,ti OR 'intra-abdominal fat':ab,ti OR 'abdominal fat':ab,ti OR 'glycemic control':ab,ti OR 'fasting glucose':ab,ti OR 'fasting blood glucose':ab,ti OR fpg:ab,ti OR 'impaired fasting glucose':ab,ti OR 'blood glucose':ab,ti OR 'glycated hemoglobin a':ab,ti OR hba1c:ab,ti OR 'hemoglobin a1c':ab,ti OR 'glycated hemoglobin':ab,ti) AND ('randomized controlled trial':ab,ti OR 'controlled clinical trial':ab,ti)	936

Appendix 1.4 Search strategy in CNKI (N = 424)

Search strategy	Results
Su =“糖尿病前期”+“糖尿病前期状态”+“糖耐量受损”+“空腹血糖受损”+“糖代谢异常”+“肥胖”+“肥胖者”+“超重”+“内脏脂肪堆积”+“腹部肥胖”+“中心型肥胖”+“腰围”+“代谢综合征”* Su =“运动”+“锻炼”+“身体活动”+“身体活跃”+“训练”+“有氧运动”+“力量训练”+“抗阻训练”+“高强度间歇训练”+“步行”+“慢跑”+“游泳”+“骑自行车”+“身心锻炼”+“太极”+“气功”+“八段锦”+“五禽戏”+“易筋经”+“瑜伽”+“中国传统武术”+“武术”* Su =“腹部脂肪”+“腹腔内脂肪”+“内脏脂肪”+“血糖”+“空腹血糖”+“FBG”+“空腹血糖受损”+“糖化血红蛋白”+“HbA1c”	424

Appendix 1.5 Search strategy in VIP (N = 78)

Search strategy	Results
(M= 糖尿病前期 OR 糖尿病前期状态 OR 糖耐量受损 OR 空腹血糖受损 OR 糖代谢异常 OR 肥胖 OR 肥胖者 OR 超重 OR 内脏脂肪堆积 OR 腹部肥胖 OR 中心型肥胖 OR 腰围 OR 代谢综合征) AND (M= 运动 OR 锻炼 OR 身体活动 OR 身体活跃 OR 训练 OR 有氧运动 OR 力量训练 OR 抗阻训练 OR 高强度间歇训练 OR 步行 OR 慢跑 OR 游泳 OR 骑自行车 OR 身心锻炼 OR 太极 OR 气功 OR 八段锦 OR 五禽戏 OR 易筋经 OR 瑜伽 OR 中国传统武术 OR 武术) AND (M= 腹部脂肪 OR 腹腔内脂肪 OR 内脏脂肪 OR 血糖 OR 空腹血糖 OR FPG OR 糖化血红蛋白 OR HbA1c)	78

Appendix 1.6 Search strategy in WANFANG (N = 549)

Search strategy	Results
题名或关键词:(糖尿病前期 OR 糖耐量受损 OR 空腹血糖受损 OR 糖代谢异常 OR 肥胖 OR 肥胖者 OR 超重 OR 内脏脂肪堆积 OR 腹部肥胖 OR 中心型肥胖 OR 腰围) and 题名或关键词:(锻炼 OR 运动 OR 身体活动 OR 身体活跃 OR 训练 OR 有氧运动 OR 力量训练 OR 抗阻训练 OR 高强度间歇训练 OR 步行 OR 慢跑 OR 游泳 OR 骑自行车 OR 身心锻炼 OR 太极) and 题名或关键词:(腹部脂肪 OR 腹腔内脂肪 OR 内脏脂肪 OR 血糖 OR 空腹血糖 OR 空腹血糖 OR FPG OR 空腹血糖受损 OR 糖化血红蛋白 OR HbA1c)	549

Appendix 2 Definitions of exercise interventions

Type of exercise	Definition
Aerobic training (AT)	Running, Walking, Aerobic dance, Swimming, Cycling, Stair climbing
Resistance training (RT)	Machine-based resistance training, Resistance band training, Bodyweight resistance training
Combined aerobic and resistance training (CT)	Combined jogging and bodyweight training, Combined aerobic training (walking/running/swimming) with resistance band training
High-Intensity Interval Training (HIIT)	HIIT cycling, HIIT swimming, HIIT with bodyweight exercises
Mind-Body Exercise (MBE)	Pilates, Traditional Thai Exercise (Ruesi Dadton)
Control Group (CON)	No exercise, Low-intensity active control

Appendix 3 Transformation formulas by exercise type

Appendix 3.1 Transformation formulas (Higgins et al. 2019)

when the sample size in each group > 100

Calculate SD through 95% confidence interval: **SE= × (upper limit − lower limit) ⁄ 3.92**

SD = SE × √N

When the sample size in each group ≤ 100

Input *t* = tinv (1-0.95, *N*−1) in Microsoft Excel to obtain t

SE= (upper limit-lower limit) / (2 × t)

SD = SE × √N

Appendix 3.2 Combined formula (Higgins et al. 2019)

The formula used to combine the data of two groups: Group 1 (Sample size = N_1, Mean = M_1 and SD = SD_1) and Group 2 (Sample size = N_2, Mean = M_2 and SD = SD_2)

$$N_{\text{combined}} = N_1 + N_2$$

$$M_{\text{combined}} = \frac{N_1 M_1 + N_2 M_2}{N_1 + N_2}$$

$$SD_{\text{combined}} = \sqrt{\frac{(N_1 - 1)SD_1^2 + (N_2 - 1)SD_2^2 + \frac{N_1 N_2}{N_1 + N_2}(M_1^2 + M_2^2 - 2M_1 M_2)}{N_1 + N_2 - 1}}$$

M = mean; SE = standard error of mean; SD = standard deviation; N = sample size.

Appendix 4 Forest plot comparing exercise versus control for VAT, FBG, and HbA1c.

VAT

Study or Subgroup	Experimental Mean	Experimental SD	Experimental Total	Control Mean	Control SD	Control Total	Weight	Std. Mean Difference IV, Random, 95% CI
Ballin 2019	-163	784.74	36	-79	1,008.66	36	6.7%	-0.09 [-0.55, 0.37]
Bouchonville 2014	-115	244	26	15	589	27	5.6%	-0.28 [-0.82, 0.26]
Chiang 2019	-12.73	17.52	11	-1	30.23	9	2.8%	-0.47 [-1.36, 0.43]
Dai 2014	-1.48	0.33	10	-0.12	0.35	10	1.0%	-3.83 [-5.42, -2.24]
Friedenreich 2011	-16.5	19.68	160	-1.6	24.2	160	11.0%	-0.67 [-0.90, -0.45]
Guo 2021	-4.16	23.81	60	0.86	25	60	8.4%	-0.20 [-0.56, 0.15]
Haganes 2022	-8	51.2	26	-5.3	49.32	29	5.7%	-0.05 [-0.58, 0.48]
Herzig 2014	-7.8	30.63	33	-2.3	30.62	35	6.5%	-0.18 [-0.65, 0.30]
Irwin 2003	-8.5	30.7	87	0.1	30.99	86	9.5%	-0.28 [-0.58, 0.02]
Jadhav 2023	-1.88	5.05	71	-0.38	4.39	67	8.8%	-0.31 [-0.65, 0.02]
Keating 2017	-175	1,366.15	15	10	1,287.15	14	3.8%	-0.14 [-0.86, 0.59]
lin 2016	-0.6	2.01	18	0	1.85	18	4.4%	-0.30 [-0.96, 0.35]
Ratajczak 2024	-36	479.91	19	13	446.76	8	3.1%	-0.10 [-0.93, 0.73]
Ross 2004	-0.7	0.75	17	-0.1	0.9	10	3.2%	-0.72 [-1.53, 0.09]
Tan 2025	-327.5	379.7	10	76.6	786.53	10	2.7%	-0.63 [-1.53, 0.28]
Tong 2018	-9.8	29.38	16	-0.2	20.42	14	3.8%	-0.36 [-1.09, 0.36]
Yan 2019(A)	-19.86	54.47	35	5.4	54.25	35	6.5%	-0.46 [-0.93, 0.02]
Yan 2019(B)	-12.74	61.51	35	5.4	54.25	35	6.5%	-0.31 [-0.78, 0.16]
**Total (95% CI)**			**685**			**663**	**100.0%**	**-0.36 [-0.53, -0.19]**

Heterogeneity: Tau² = 0.05; Chi² = 32.07, df = 17 (P = 0.01); I² = 47%
Test for overall effect: Z = 4.24 (P < 0.0001)

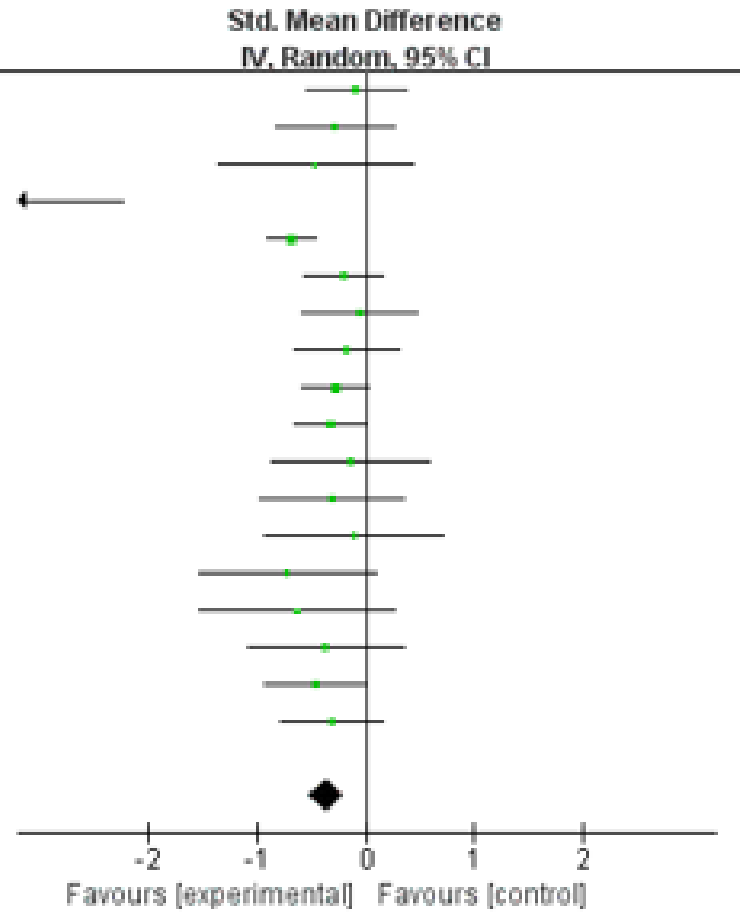

FBG

Study or Subgroup	Experimental Mean	Experimental SD	Experimental Total	Control Mean	Control SD	Control Total	Weight	Mean Difference IV, Random, 95% CI
Bouchonville 2014	-0.07	0.46	26	-0.03	0.66	27	3.7%	-0.04 [-0.35, 0.27]
Chen 2021(A)	-0.22	1.48	83	0.4	2.18	83	2.6%	-0.62 [-1.19, -0.05]
Chen 2021(B)	-0.3	1.75	82	0.4	2.18	83	2.4%	-0.70 [-1.30, -0.10]
Chow 2020	-0.15	0.4	21	-0.01	0.7	10	3.0%	-0.14 [-0.61, 0.33]
Hafeez 2025	-0.67	0.14	72	-0.01	0.11	36	4.6%	-0.66 [-0.71, -0.61]
Haganes 2022	-0.1	0.46	26	0	0.4	28	4.1%	-0.10 [-0.33, 0.13]
Han 2022	-0.34	0.51	58	0.15	0.5	55	4.2%	-0.49 [-0.68, -0.30]
Herzig 2014	-0.2	0.8	33	0.1	1.57	35	2.5%	-0.30 [-0.89, 0.29]
Ho 2012(A)	0.05	0.57	15	-0.09	0.64	16	3.2%	0.14 [-0.29, 0.57]
Ho 2012(B)	-0.04	1.62	16	-0.09	0.64	16	1.6%	0.05 [-0.80, 0.90]
Ho 2012(C)	0.17	0.54	17	-0.09	0.64	16	3.3%	0.26 [-0.15, 0.67]
Jadhav 2023	-0.46	0.72	71	-0.16	0.46	67	4.2%	-0.30 [-0.50, -0.10]
Karaman 2025	-0.82	0.69	21	0.22	1.61	21	1.9%	-1.04 [-1.79, -0.29]
Luo 2023(A)	-0.41	0.53	26	0.02	0.6	21	3.6%	-0.43 [-0.76, -0.10]
Luo 2023(B)	-0.31	0.7	23	0.02	0.6	21	3.4%	-0.33 [-0.71, 0.05]
Morey 2012	-0.34	0.48	180	-0.35	0.58	122	4.4%	0.01 [-0.11, 0.13]
Ratajczak 2024	0	0.46	19	-0.15	0.26	8	3.9%	0.15 [-0.12, 0.42]
Ross 2004	-0.32	0.58	17	-0.25	0.54	10	3.1%	-0.07 [-0.50, 0.36]
Sawangwong 2023	-1.05	0.42	31	0.14	0.45	30	4.1%	-1.19 [-1.41, -0.97]
Shahiddoust 2025(A)	-0.18	0.5	9	-0.06	0.46	10	3.1%	-0.12 [-0.55, 0.31]
Shahiddoust 2025(B)	-0.17	0.26	10	-0.06	0.46	10	3.6%	-0.11 [-0.44, 0.22]
Siu 2025	-0.1	0.93	210	0	1.3	105	3.8%	-0.10 [-0.38, 0.18]
Streb 2021	-0.19	0.72	46	-0.01	1.01	23	3.0%	-0.18 [-0.64, 0.28]
Tomeleri 2016	-0.32	0.64	19	0.18	0.44	19	3.5%	-0.50 [-0.85, -0.15]
Wang 2013	-0.26	0.6	64	0.17	0.5	18	3.9%	-0.43 [-0.70, -0.16]
Yan 2019(A)	-0.19	0.65	35	0.28	0.81	35	3.5%	-0.47 [-0.81, -0.13]
Yan 2019(B)	-0.17	0.72	35	0.28	0.81	35	3.5%	-0.45 [-0.81, -0.09]
Yuan 2020(A)	-0.2	0.61	83	0.29	0.68	83	4.2%	-0.49 [-0.69, -0.29]
Yuan 2020(B)	-0.33	0.6	82	0.29	0.68	83	4.2%	-0.62 [-0.82, -0.42]
**Total (95% CI)**			**1430**			**1126**	**100.0%**	**-0.32 [-0.46, -0.18]**

Heterogeneity: Tau² = 0.11; Chi² = 244.21, df = 28 (P < 0.00001); I² = 89%
Test for overall effect: Z = 4.62 (P < 0.00001)

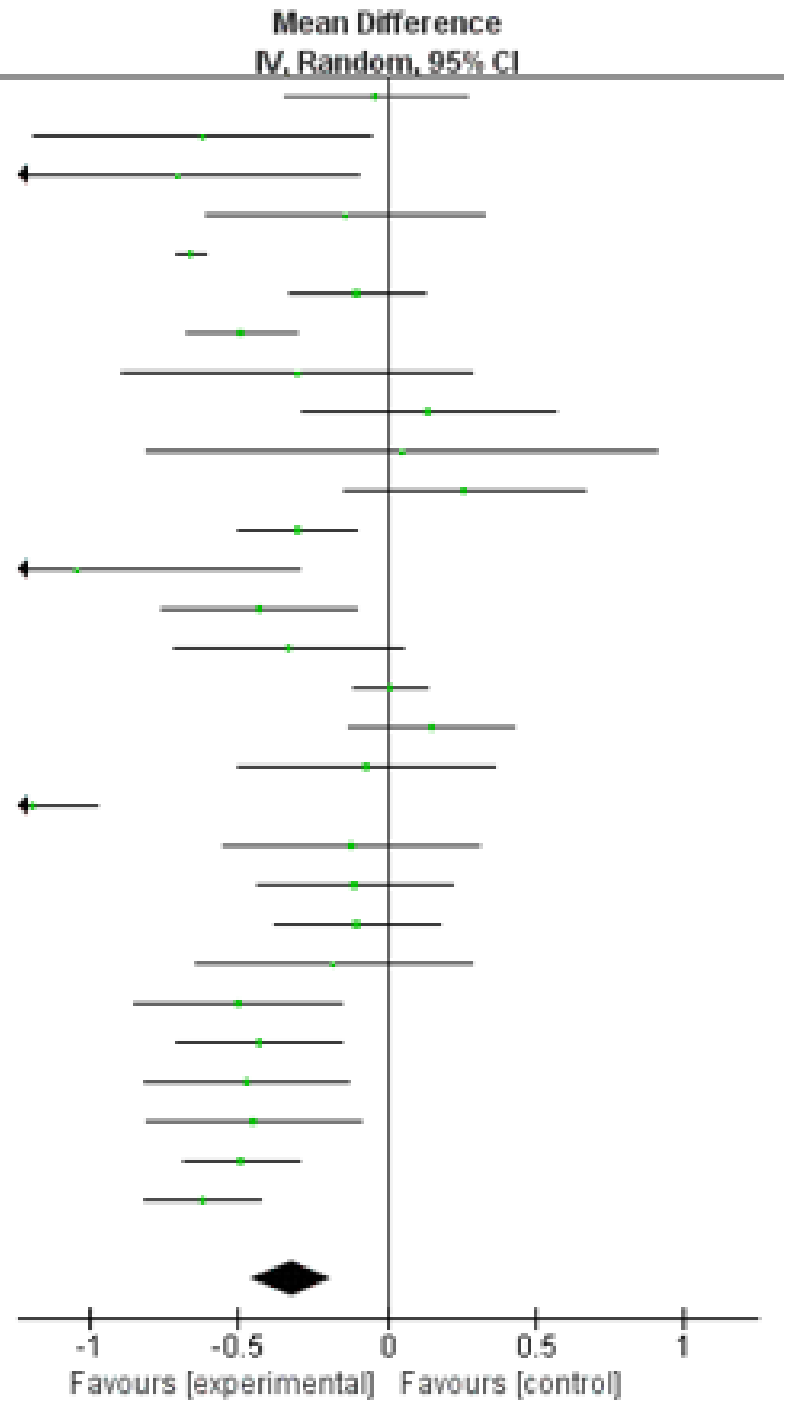

HbA1c

Study or Subgroup	Experimental Mean	Experimental SD	Experimental Total	Control Mean	Control SD	Control Total	Weight	Mean Difference IV, Random, 95% CI
Chen 2021(A)	-0.3	1.27	83	0.3	1.82	83	4.5%	-0.60 [-1.08, -0.12]
Chen 2021(B)	-0.1	1.52	82	0.3	1.82	83	4.2%	-0.40 [-0.91, 0.11]
Hafeez 2025	-0.69	0.17	72	-0.14	0.18	36	10.1%	-0.55 [-0.62, -0.48]
Haganes 2022	-0.02	0.35	26	0.04	0.27	27	9.0%	-0.06 [-0.23, 0.11]
Jadhav 2023	-0.43	0.3	71	0	0.32	67	9.8%	-0.43 [-0.53, -0.33]
Karaman 2025	-0.3	0.37	21	0.1	0.35	21	8.2%	-0.40 [-0.62, -0.18]
Morey 2012	0.01	0.43	180	0.02	0.39	122	9.9%	-0.01 [-0.10, 0.08]
Sawangwong 2023	-0.23	0.36	31	0.07	0.41	30	8.6%	-0.30 [-0.49, -0.11]
Yan 2019(A)	-0.15	0.37	35	0.06	0.57	35	8.1%	-0.21 [-0.44, 0.02]
Yan 2019(B)	-0.05	0.35	35	0.06	0.57	35	8.2%	-0.11 [-0.33, 0.11]
Yuan 2020(A)	-0.05	0.39	83	0.2	0.41	83	9.6%	-0.25 [-0.37, -0.13]
Yuan 2020(B)	0.05	0.32	82	0.2	0.41	83	9.7%	-0.15 [-0.26, -0.04]
**Total (95% CI)**			**801**			**705**	**100.0%**	**-0.27 [-0.41, -0.14]**

Heterogeneity: Tau² = 0.05; Chi² = 112.19, df = 11 (P < 0.00001); I² = 90%
Test for overall effect: Z = 3.93 (P < 0.0001)

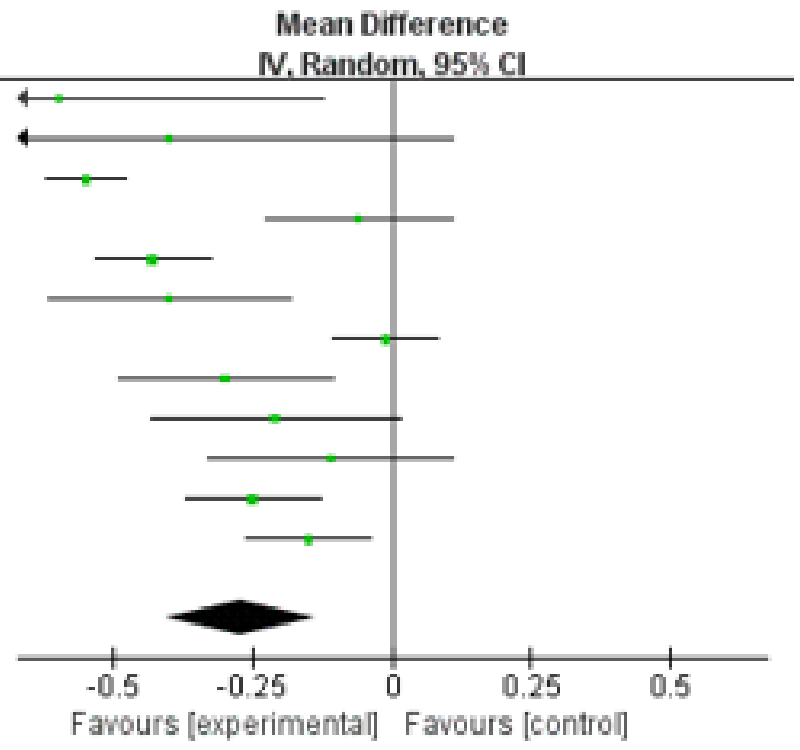

Appendix 5 Prediction interval forest plots for VAT, FBG and HbA1c

Appendix 5.1 Prediction interval forest plots for VAT

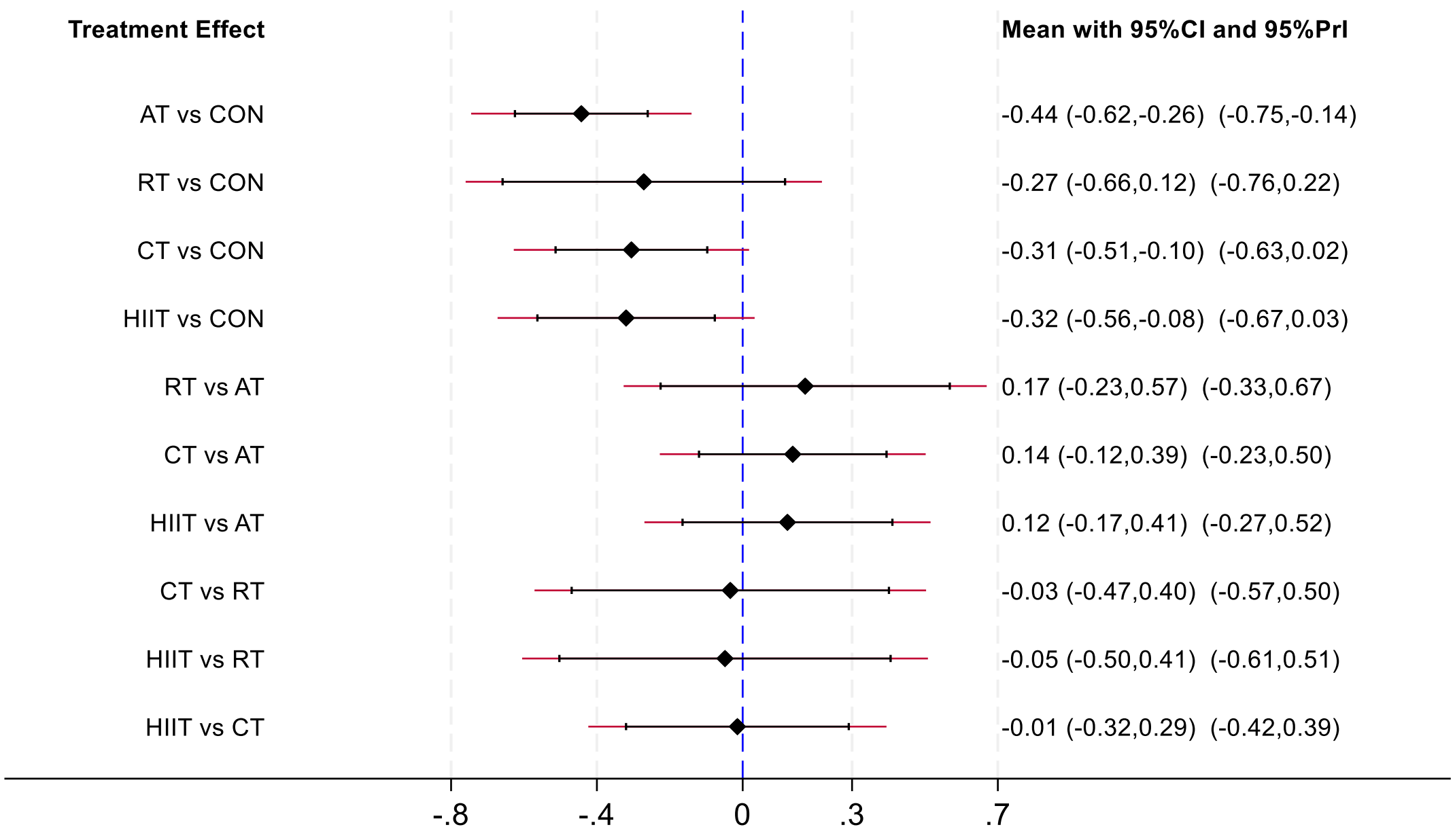

Note. AT: aerobic training; RT: resistance training; CT: combined aerobic and resistance training; HIIT: high-intensity interval training; MBE: mind-body exercise; CON: control group.

Appendix 5.2 Prediction interval forest plots for FBG

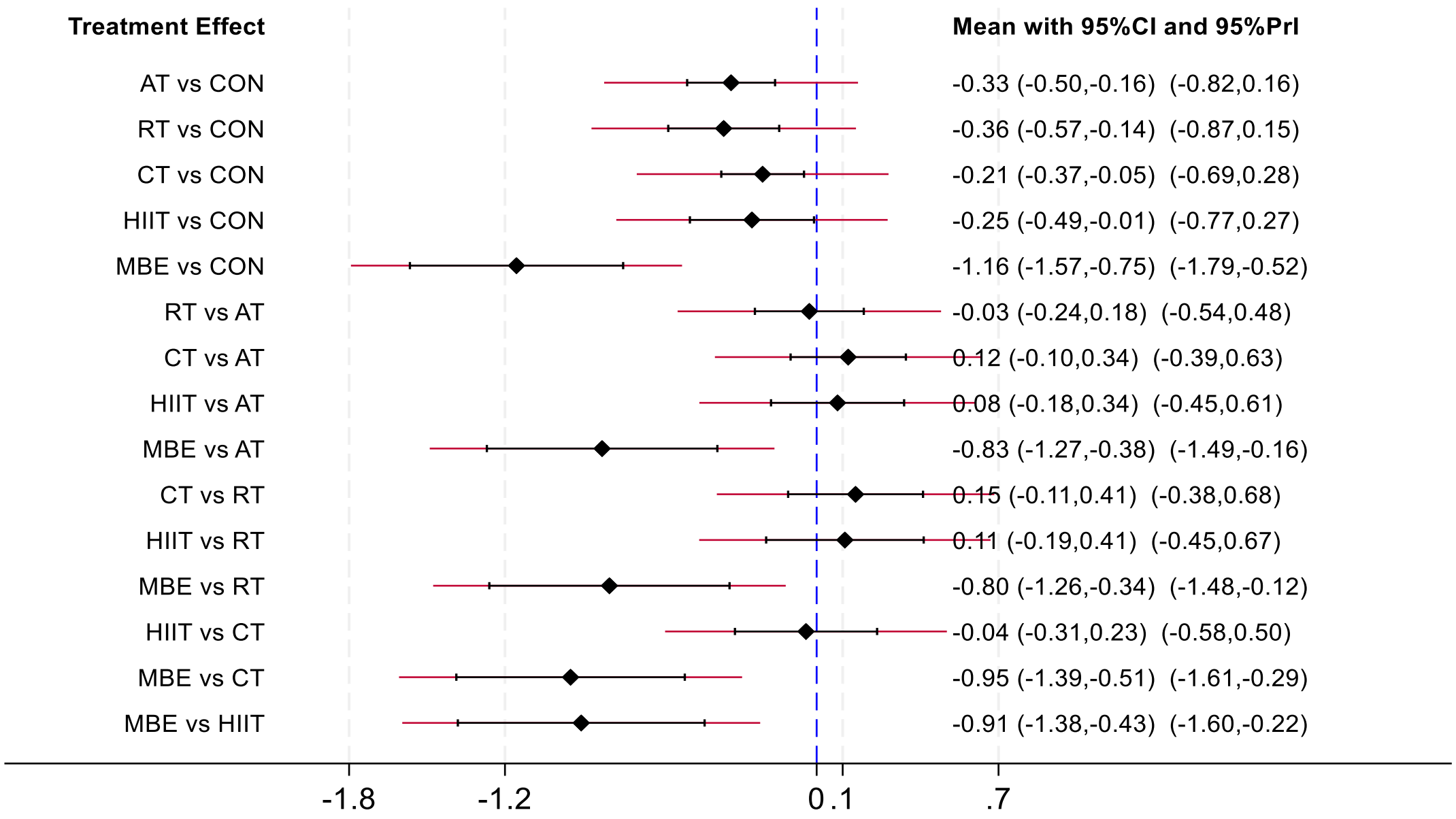

Appendix 5.3 Prediction interval forest plots for HbA1c

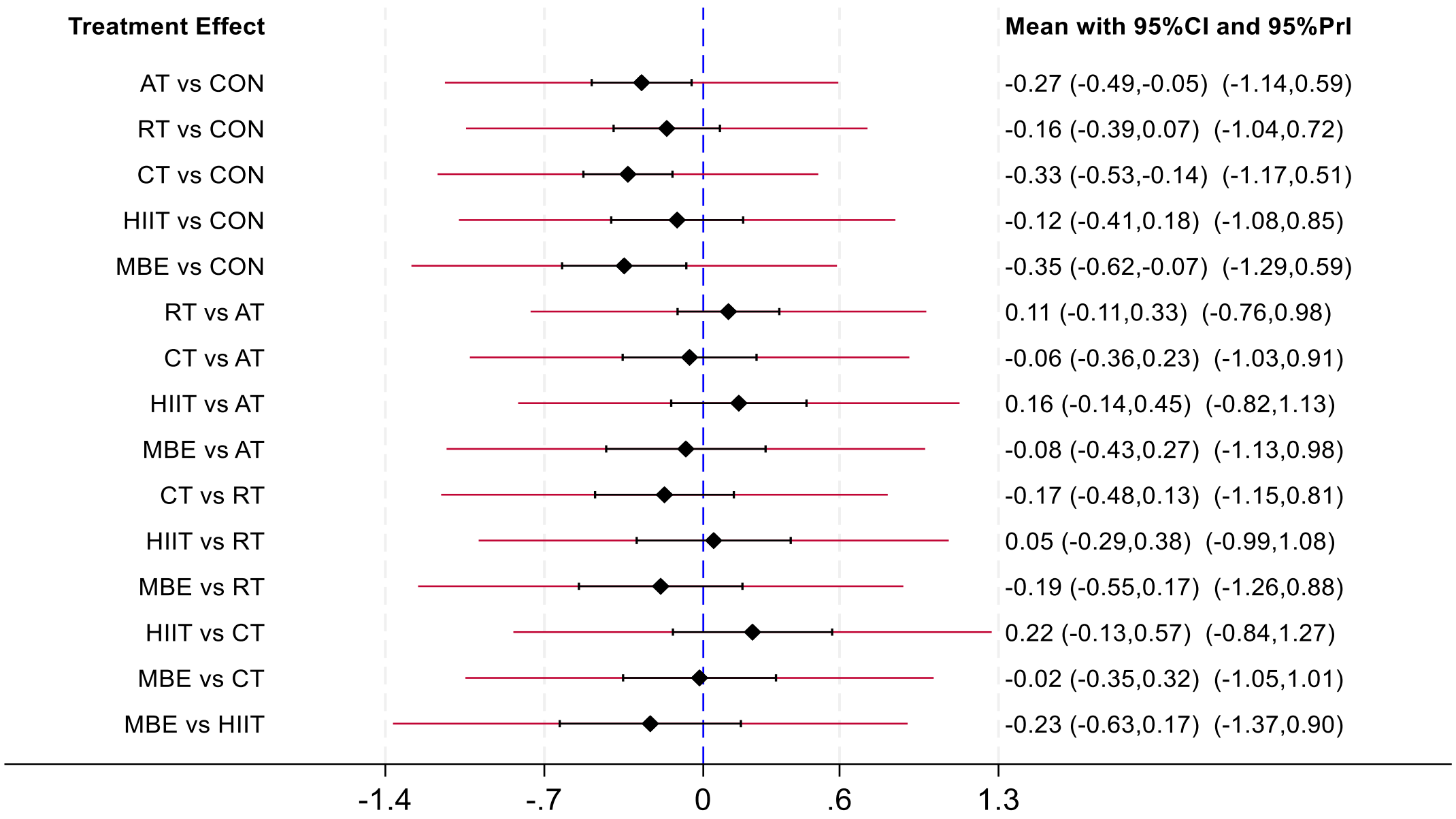

Appendix 6 Inconsistency Models, Loop-Specific Heterogeneity, and Node-Splitting Analyses for VAT, FBG and HbA1c.

Appendix 6.1 Inconsistency model for VAT, FBG and HbA1c

	VAT	FBG	HbA1c
chi2	0.95	12.13	0.21
Prob > chi2	0.97	0.21	0.64

Appendix 6.2 loop-specific heterogeneity estimates for VAT, FBG and HbA1c

Loop	IF	seIF	z_value	p_value	CI_95	Loop_Heterog_tau2
VAT						
AT-CT-HIIT	0.452	0.533	0.848	0.397	(0.00,1.50)	0
CON-AT-CT	0.295	0.29	1.02	0.308	(0.00,0.86)	0
CON-AT-HIIT	0.219	0.688	0.318	0.751	(0.00,1.57)	0.127
CON-AT-RT	0.069	0.387	0.179	0.858	(0.00,0.83)	0.023
CON-CT-HIIT	0.024	0.879	0.027	0.978	(0.00,1.75)	0.13
FBG						
CON-AT-HIIT	0.382	0.163	2.345	0.019	(0.06,0.70)	0.003
CON-AT-RT	0.272	0.163	1.669	0.095	(0.00,0.59)	0.009
AT-CT-HIIT	0.223	0.293	0.76	0.448	(0.00,0.80)	0
AT-RT-CT	0.156	0.551	0.284	0.777	(0.00,1.24)	0.035
CON-CT-HIIT	0.077	0.496	0.156	0.876	(0.00,1.05)	0.137
CON-RT-CT	0.039	0.591	0.065	0.948	(0.00,1.20)	0.122
CON-AT-CT	0.014	0.433	0.033	0.973	(0.00,0.86)	0.115
HbA1c						
CON-AT-HIIT	0.11	0.172	0.643	0.52	(0.00,0.45)	0.001
CON-AT-RT	-	-	-	-	-	0

Appendix 6.3 Node splitting analysis for VAT, FBG and HbA1c

Side	Direct Coef.	Std. Err.	Indirect Coef.	Std. Err.	Difference Coef.	Std. Err.	P>z	tau
				VAT				
CON: AT	-0.46	0.10	-0.35	0.21	-0.12	0.24	0.62	0.10
CON: RT*	-0.26	0.22	-0.32	0.50	0.06	0.55	0.91	0.11
CON: CT	-0.28	0.12	-0.42	0.25	0.14	0.27	0.61	0.11
CON: HIIT	-0.33	0.15	-0.31	0.24	-0.02	0.29	0.94	0.11
AT: RT	0.12	0.26	0.24	0.33	-0.12	0.42	0.78	0.11
AT: CT	-0.08	0.28	0.20	0.15	-0.28	0.31	0.37	0.10
AT: HIIT	0.20	0.28	0.08	0.18	0.12	0.33	0.71	0.12
CT: HIIT	-0.17	0.41	0.01	0.17	-0.18	0.44	0.69	0.10
				FBG				
CON: AT	-0.33	0.10	-0.33	0.20	0.00	0.22	0.99	0.22
CON: RT	-0.44	0.12	-0.02	0.23	-0.43	0.26	0.10	0.20
CON: CT *	-0.19	0.08	-0.56	0.40	0.37	0.41	0.36	0.21
CON: HIIT	-0.11	0.15	-0.50	0.20	0.40	0.25	0.11	0.20
CON: MBE	-	-	-	-	-	-	-	-
AT: RT	0.04	0.11	-0.40	0.26	0.44	0.28	0.11	0.20
AT: CT	0.13	0.29	0.12	0.12	0.01	0.32	0.96	0.22
AT: HIIT	-0.16	0.20	0.26	0.17	-0.41	0.26	0.11	0.20
RT: CT	0.39	0.40	0.12	0.14	0.27	0.43	0.52	0.21
CT: HIIT	-0.03	0.28	-0.05	0.16	0.02	0.32	0.95	0.22
HbA1c								
CON: AT *	-0.30	0.13	-0.15	0.29	-0.15	0.32	0.64	0.18
CON: RT *	-0.18	0.13	0.12	0.62	-0.29	0.64	0.64	0.18
CON: CT	-	-	-	-	-	-	-	-
CON: HIIT	-0.06	0.20	-0.21	0.25	0.15	0.32	0.64	0.18
CON: MBE	-	-	-	-	-	-	-	-
AT: RT *	0.12	0.12	-0.17	0.63	0.29	0.64	0.64	0.18
AT: HIIT	0.09	0.22	0.24	0.24	-0.15	0.32	0.64	0.18

Appendix 7 The funnel plot graphics of VAT, FBG and HbA1c in NMA

Appendix 7.1 The funnel plot graphics of VAT

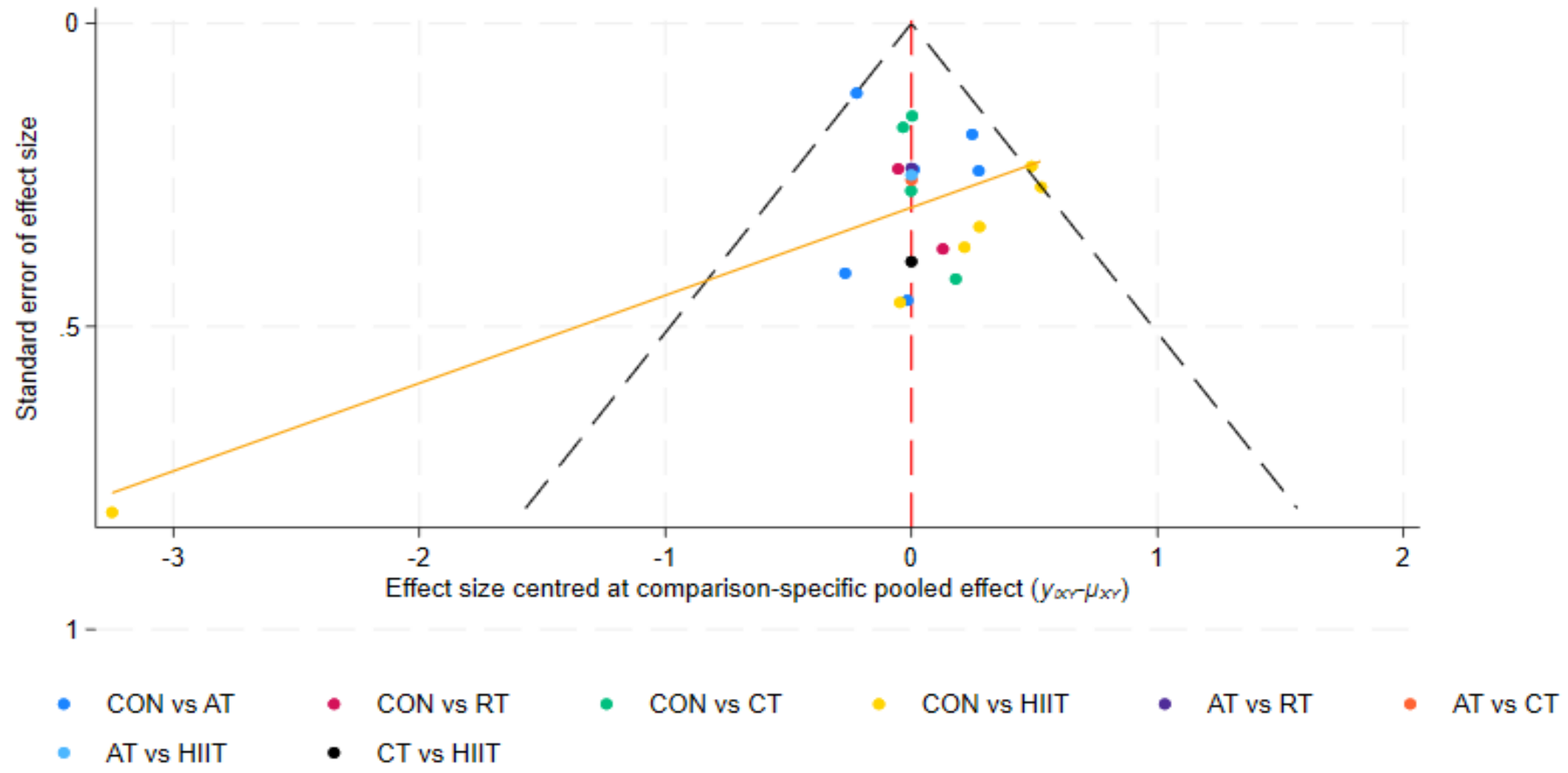

Appendix 7.2 The funnel plot graphics of FBG

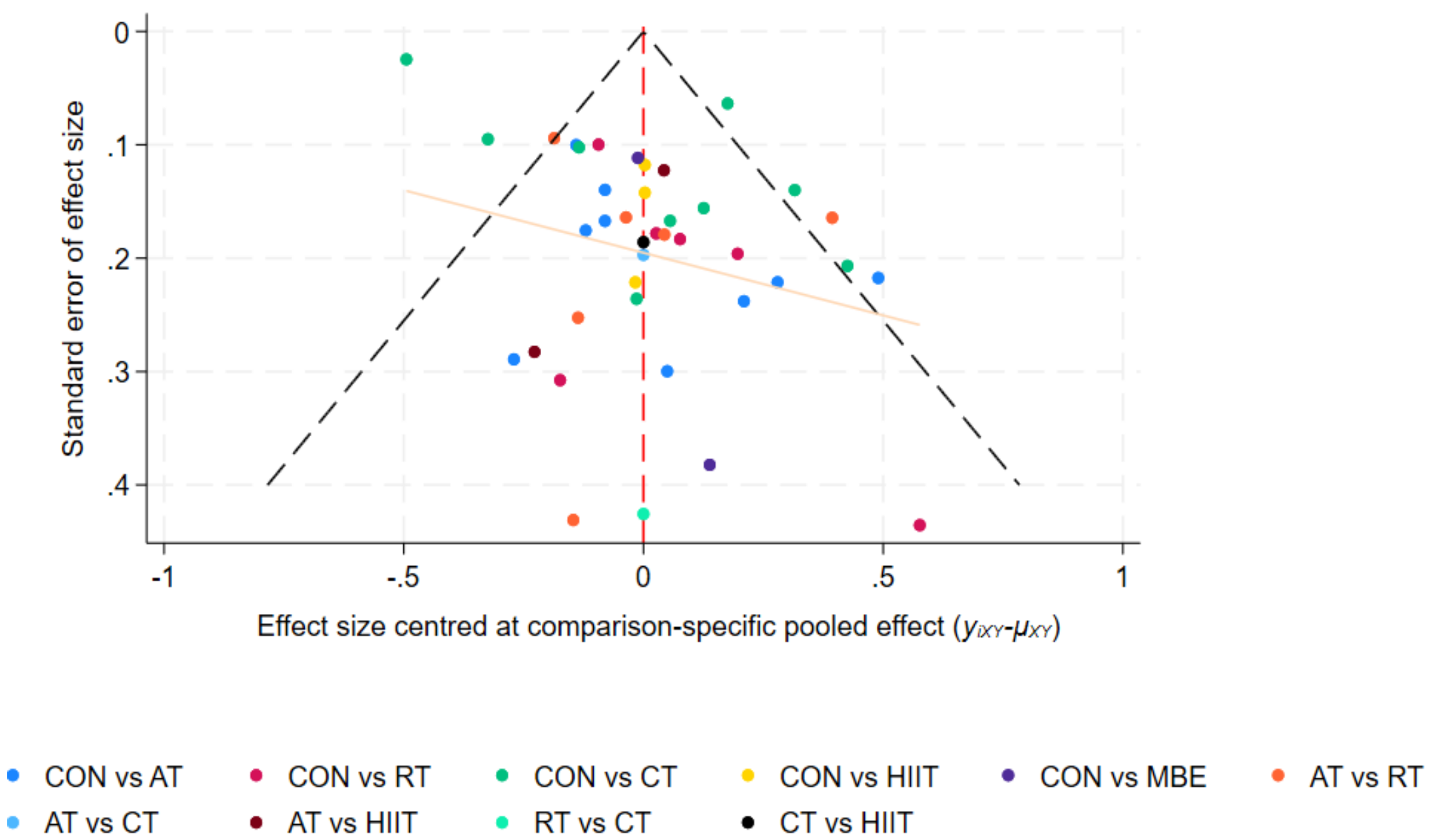

Appendix 7.3 The funnel plot graphics of HbA1c

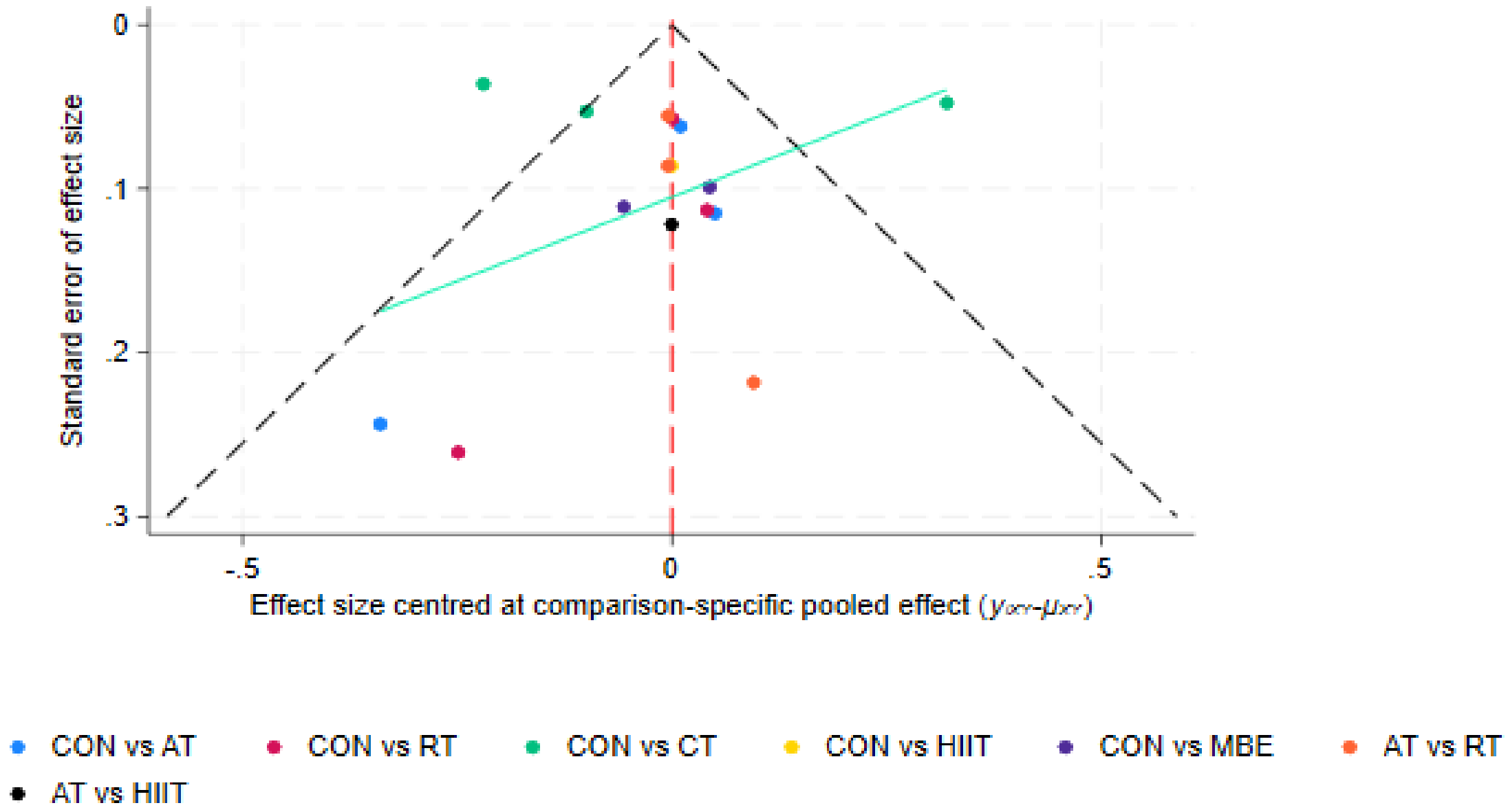

Appendix 8 Measurement methods for different outcome variables

Outcome measures	Measurement method
VAT	Bioelectrical Impedance Analysis (BIA)
	Computed Tomography (CT)
	Magnetic Resonance Imaging (MRI)
	Dual-energy X-ray absorptiometry (DXA)
FBG	Enzymatic methods
	NA
HbA1c	Immunochemical assay
	Chromatography
	NA

Appendix 9 Risk assessment of Bias in the included Studies

Appendix 9.1 The overall risk of bias for all included studies

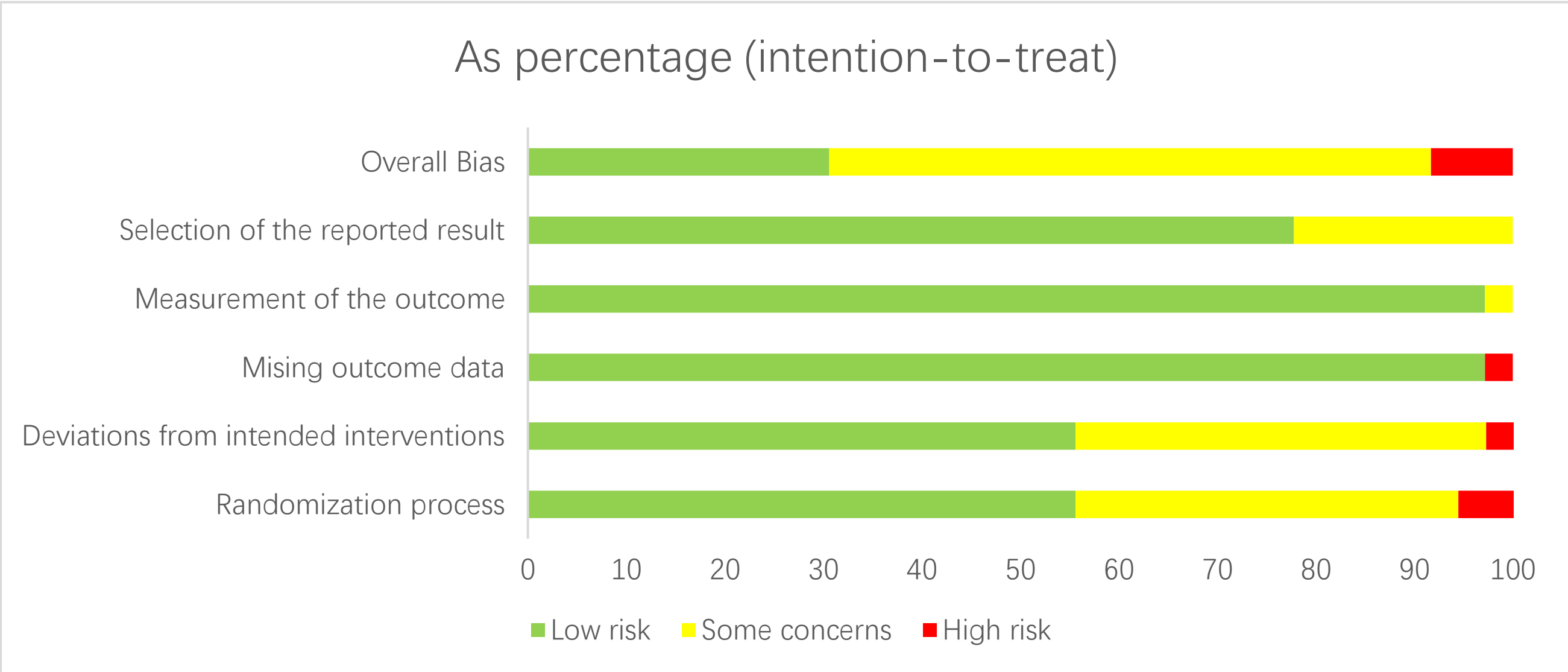

Appendix 9.2 The risk of bias for each study

Study ID	D1	D2	D3	D4	D5	Overall
Guo 2021	!	+	+	+	!	!
lin 2016	!	+	+	+	!	!
Dai 2014	!	+	+	+	!	!
Qi 2020	!	+	+	+	!	!
Tong 2018	+	+	+	+	+	+
Chiang 2019	+	+	+	+	+	+
Ross 2004	-	!	+	+	+	-
Haganes 2022	+	+	+	+	+	+
Friedenreich 2011	+	!	+	+	+	!
Nunes 2019	+	!	+	+	+	!
Ballin 2019	+	!	+	+	+	!
Keating 2017	+	+	+	+	+	+
Han 2022	!	!	+	+	!	!
Wu 2018	+	+	+	+	!	!
Yan 2019	!	!	+	+	+	!
Jadhav 2023	+	!	+	!	+	!
Luo 2023	!	+	+	+	+	!
Karaman 2025	!	+	+	+	+	!
Chen 2025	+	+	+	+	+	+
Morey 2012	+	!	+	+	+	!
Herzig 2014	!	!	+	+	+	!
Hafeez 2025	!	!	+	+	+	!
Wang 2013	-	!	+	+	!	-
Irwin 2003	+	+	+	+	+	+
Chow 2020	!	+	+	+	!	!
Chen 2021	!	!	+	+	+	!
Bouchonville 2014	+	!	+	+	+	!
Tomeleri 2016	!	+	+	+	!	!
Streb 2021	+	!	+	+	+	!
Ratajczak 2024	+	-	-	+	+	-
Yuan 2020	+	+	+	+	+	+
Sawangwong 2023	!	+	+	+	+	!
Siu 2025	+	+	+	+	+	+
Shahiddoust 2025	+	+	+	+	+	+
Ho 2012	!	!	+	+	+	!
Strijcker 2018	+	+	+	+	+	+
Tan 2025	+	+	+	+	+	+

+ Low risk
! Some concerns
- High risk

D1 Randomisation process
D2 Deviations from the intended interventions
D3 Missing outcome data
D4 Measurement of the outcome
D5 Selection of the reported result

Appendix 10 Results of the CINeMA assessment for VAT, FBG and HbA1c

Appendix 10.1 Results of the CINeMA assessment for VAT

Comparison	Number of studies	Within-study bias	Reporting bias	Indirectness	Imprecision	Heterogeneity	Incoherence	Confidence rating
CON: AT	6	Some concerns	Low risk	No concerns	No concerns	Some concerns	No concerns	Low
CON: RT	1	Some concerns	Low risk	No concerns	Major concerns	No concerns	No concerns	Low
CON: CT	5	Some concerns	Low risk	No concerns	No concerns	Major concerns	No concerns	Low
CON: HIIT	6	Some concerns	Low risk	No concerns	No concerns	Major concerns	No concerns	Low
AT: RT	1	Some concerns	Low risk	No concerns	Major concerns	No concerns	No concerns	Low
AT: CT	1	Some concerns	Low risk	No concerns	Major concerns	No concerns	No concerns	Low
AT: HIIT	1	Some concerns	Low risk	No concerns	Major concerns	No concerns	No concerns	Low
CT: HIIT	1	Some concerns	Low risk	No concerns	Major concerns	No concerns	No concerns	Low
RT: CT	0	Some concerns	Low risk	No concerns	Major concerns	No concerns	Major concerns	Very low
RT: HIIT	0	Some concerns	Low risk	No concerns	Major concerns	No concerns	Major concerns	Very low

Appendix 10.2 Results of the CINeMA assessment for FBG

Comparison	Number of studies	Within-study bias	Reporting bias	Indirectness	Imprecision	Heterogeneity	Incoherence	Confidence rating
CON:AT	9	Some concerns	Low risk	Some concerns	No concerns	Major concerns	No concerns	Very low
CON:RT	6	Some concerns	Low risk	No concerns	No concerns	Major concerns	Some concerns	Low
CON:CT	9	Some concerns	Low risk	Some concerns	Some concerns	Some concerns	No concerns	Low
CON:HIIT	3	No concerns	Low risk	No concerns	Some concerns	Some concerns	No concerns	Low
CON:MBE	2	Some concerns	Low risk	Some concerns	No concerns	No concerns	Major concerns	Very low
AT:RT	6	Some concerns	Low risk	No concerns	Major concerns	No concerns	No concerns	Low
AT:CT	1	Some concerns	Low risk	Some concerns	Some concerns	Some concerns	No concerns	Low
AT:HIIT	2	No concerns	Low risk	No concerns	Major concerns	No concerns	No concerns	Low
RT:CT	1	Some concerns	Low risk	Some concerns	Some concerns	Some concerns	No concerns	Low
CT:HIIT	1	No concerns	Low risk	No concerns	Major concerns	No concerns	No concerns	Low
AT:MBE	0	Some concerns	Low risk	Some concerns	No concerns	Some concerns	Major concerns	Very low
RT:HIIT	0	No concerns	Low risk	No concerns	Major concerns	No concerns	Major concerns	Very low
RT:MBE	0	Some concerns	Low risk	No concerns	No concerns	Some concerns	Major concerns	Very low
CT:MBE	0	Some concerns	Low risk	Some concerns	No concerns	No concerns	Major concerns	Very low
HIIT:MBE	0	Some concerns	Low risk	No concerns	No concerns	Some concerns	Major concerns	Very low

Appendix 10.3 Results of the CINeMA assessment for HbA1c

Comparison	Number of studies	Within-study bias	Reporting bias	Indirectness	Imprecision	Heterogeneity	Incoherence	Confidence rating
CON:AT	3	No concerns	Low risk	No concerns	No concerns	Major concerns	No concerns	Low
CON:RT	3	Some concerns	Low risk	No concerns	Some concerns	Some concerns	No concerns	Low
CON:CT	3	Some concerns	Low risk	No concerns	No concerns	Major concerns	Major concerns	Very low
CON:HIIT	1	No concerns	Low risk	No concerns	Major concerns	No concerns	No concerns	Low
CON:MBE	2	Some concerns	Low risk	No concerns	No concerns	Major concerns	Major concerns	Very low
AT:RT	3	Some concerns	Low risk	No concerns	Some concerns	Some concerns	No concerns	Low
AT:HIIT	1	No concerns	Low risk	No concerns	Major concerns	No concerns	No concerns	Very low
AT:CT	0	Some concerns	Low risk	No concerns	Major concerns	No concerns	Major concerns	Very low
AT:MBE	0	Some concerns	Low risk	No concerns	Major concerns	No concerns	Major concerns	Very low
RT:CT	0	Some concerns	Low risk	No concerns	Major concerns	No concerns	Major concerns	Very low
RT:HIIT	0	No concerns	Low risk	No concerns	Major concerns	No concerns	Major concerns	Very low
RT:MBE	0	Some concerns	Low risk	No concerns	Major concerns	No concerns	Major concerns	Very low
CT:HIIT	0	Some concerns	Low risk	No concerns	Major concerns	No concerns	Major concerns	Very low
CT:MBE	0	Some concerns	Low risk	No concerns	Major concerns	No concerns	Major concerns	Very low
HIIT:MBE	0	Some concerns	Low risk	No concerns	Major concerns	No concerns	Major concerns	Very low

Appendix 11 Contributions of direct and indirect comparisons to NMA and the number of studies per comparison for VAT, FBG and HbA1c.

Appendix 11.1 VAT

Direct comparisons in the network

Network meta-analysis estimates	AvsB	AvsC	AvsD	AvsE	BvsC	BvsD	BvsE	DvsE
**Mixed estimates**								
AvsB	57.0	6.9	10.1	3.8	6.9	8.5	5.3	1.5
AvsC	20.9	42.5	3.7	1.4	25.9	3.1	1.9	0.6
AvsD	9.7	1.2	71.4	2.2	1.2	9.5	1.4	3.5
AvsE	21.7	2.6	13.1	20.7	2.6	1.8	26.1	11.4
BvsC	26.0	32.3	4.6	1.7	28.4	3.9	2.4	0.7
BvsD	33.0	4.0	38.2	1.2	4.0	11.8	4.5	3.3
BvsE	22.2	2.7	5.9	18.9	2.7	4.9	31.9	10.8
DvsE	12.4	1.5	29.7	15.8	1.5	6.9	20.8	11.4
**Indirect estimates**								
CvsD	8.5	27.1	35.3	0.3	17.5	7.0	2.0	2.2
CvsE	3.0	25.5	7.6	14.9	18.8	0.7	21.2	8.3
**Entire network**	19.9	15.2	21.4	8.5	11.3	5.6	12.4	5.7
**Included studies**	6	2	4	6	1	1	1	1

Note. A: control group; B: aerobic training; C: resistance training; D: combined aerobic and resistance training; E: high-intensity interval training.

Appendix 11.2 FBG

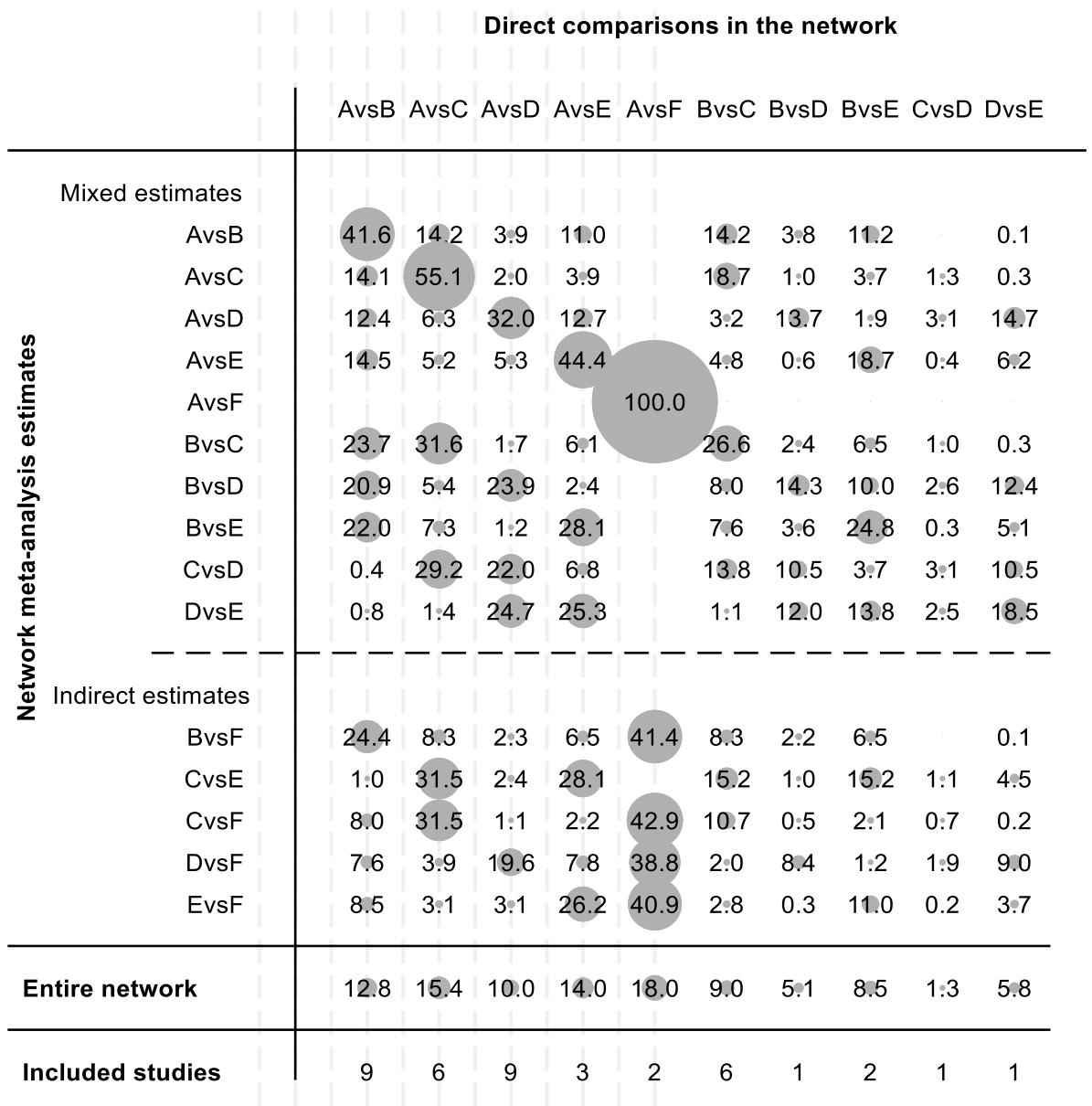

Note. A: control group; B: aerobic training; C: resistance training; D: combined aerobic and resistance training; E: high-intensity interval training; F: mind-body exercise.

Appendix 11.3 HbA1c

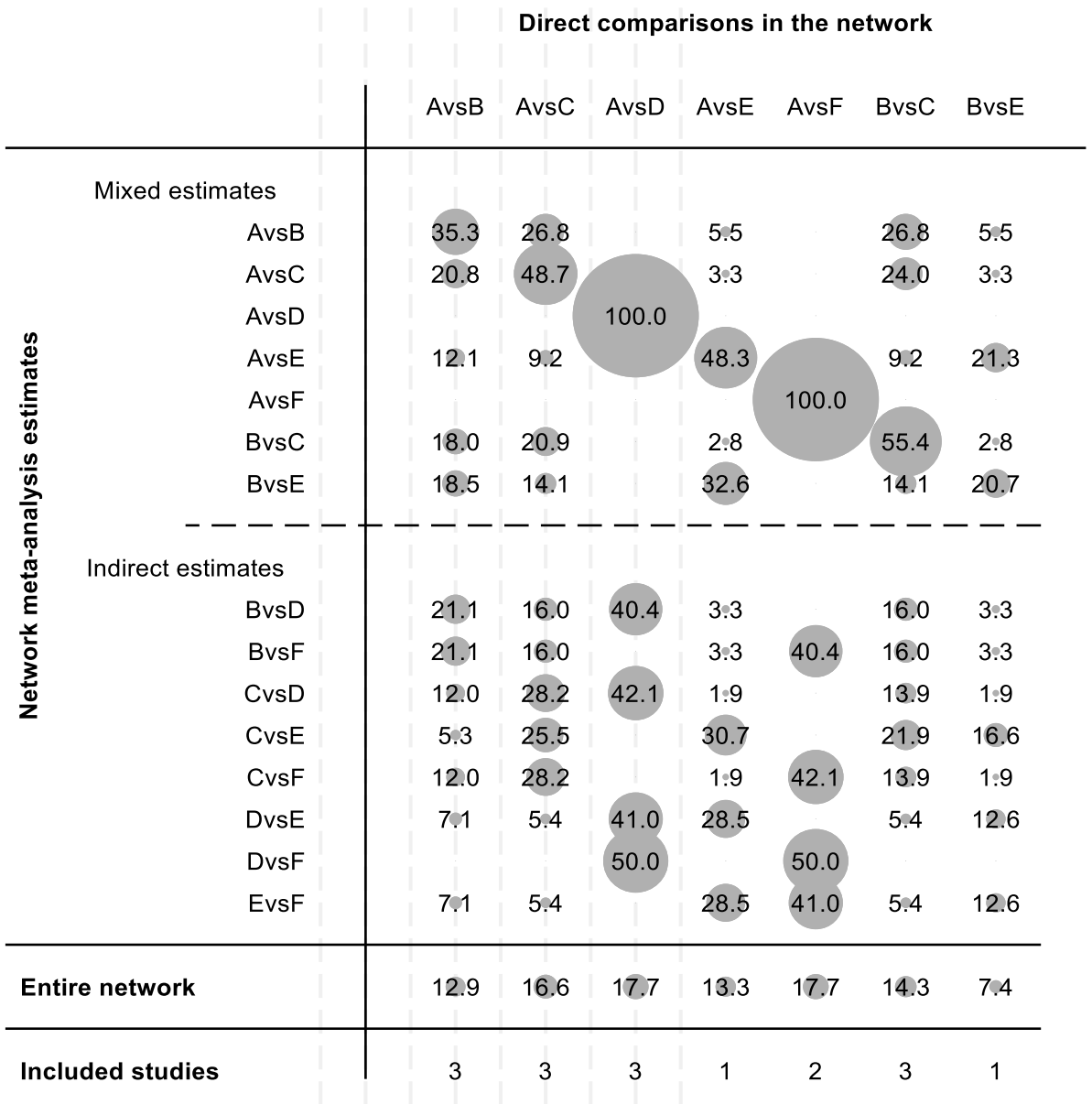

Note. A: control group; B: aerobic training; C: resistance training; D: combined aerobic and resistance training; E: high-intensity interval training; F: mind-body exercise.

Appendix 12 SUCRA ranking for VAT, FBG and HbA1c

Appendix 12.1 SUCRA ranking for VAT

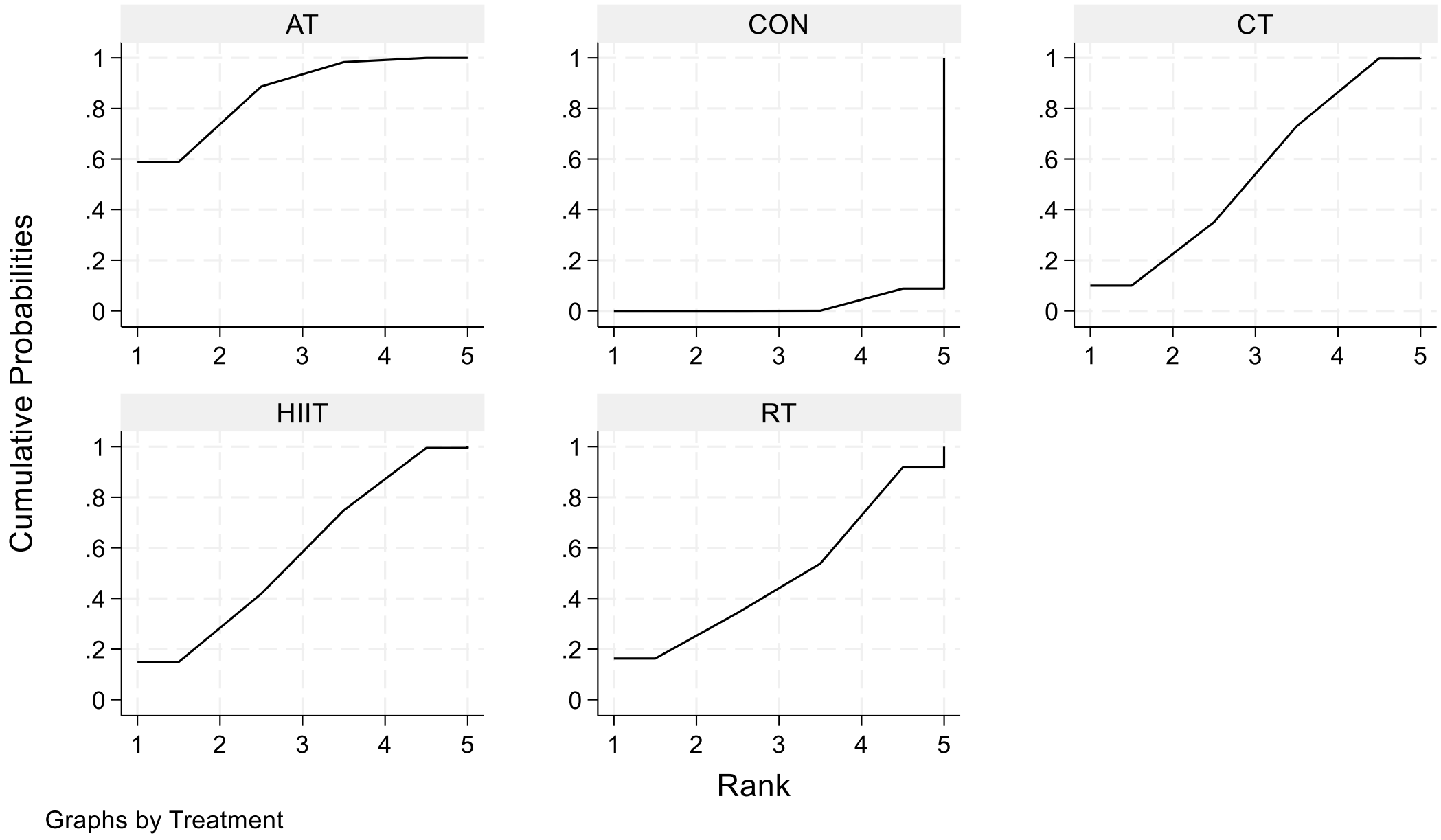

Note. AT: aerobic training; RT: resistance training; CT: combined aerobic and resistance training; HIIT: high-intensity interval training; MBE: mind-body exercise; CON: control group.

Appendix 12.2 SUCRA ranking for FBG

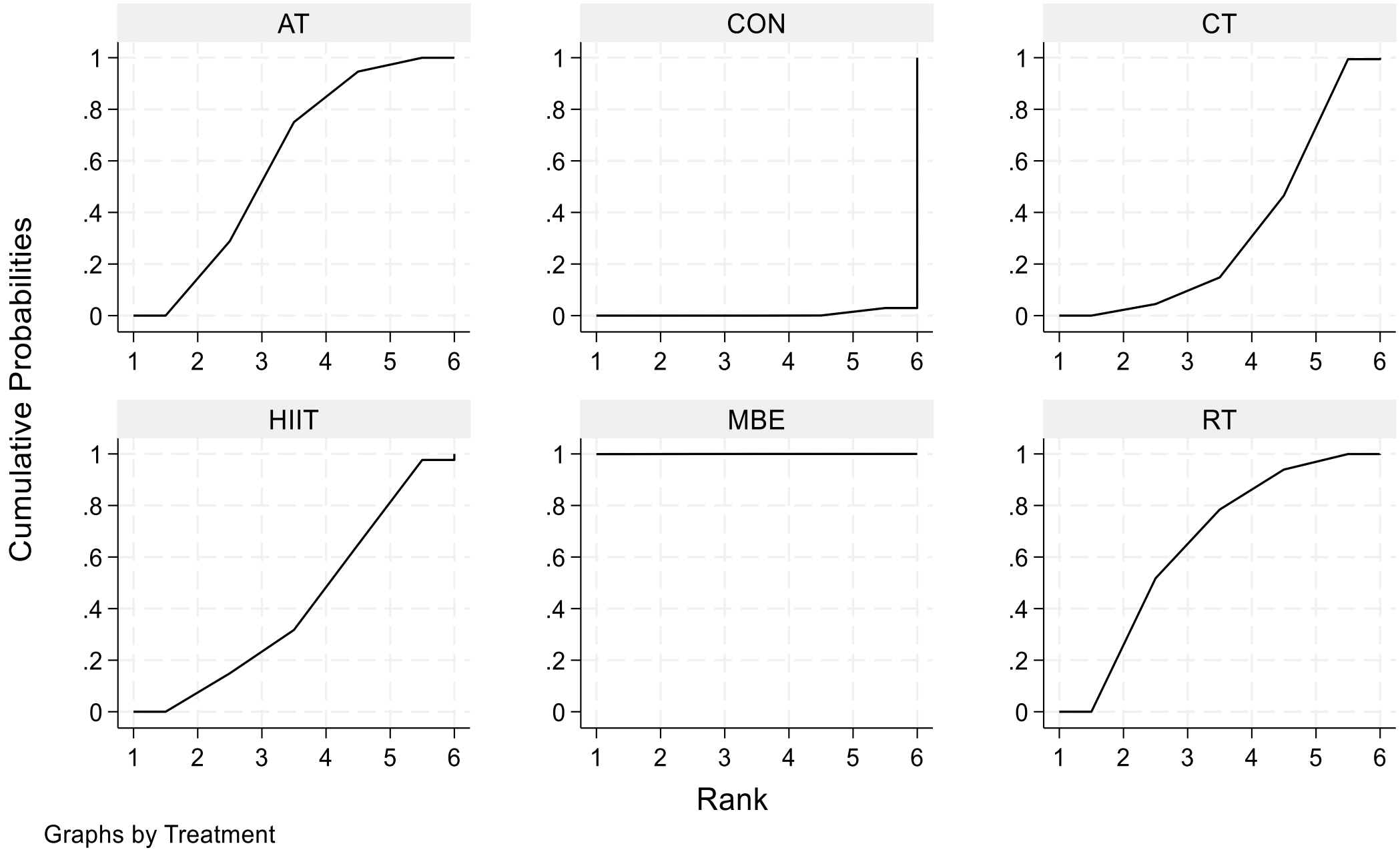

Appendix 12.3 SUCRA ranking for HbA1c

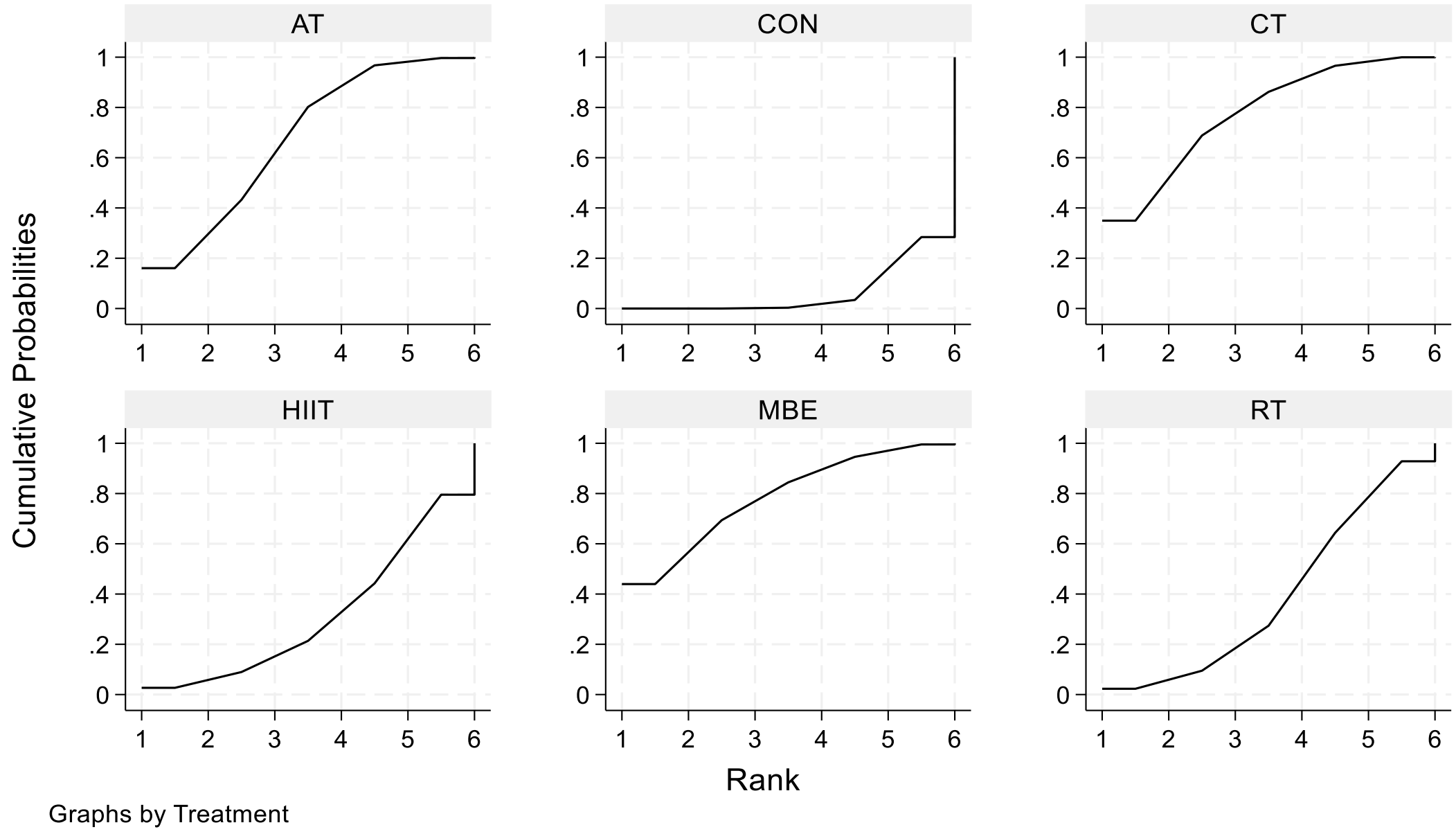

Appendix 13 Inconsistency model for subgroups of VAT and FBG

Appendix 13.1 Inconsistency model for subgroups of VAT

VAT	chi2	Prob > chi2
≤ 12 weeks	0.49	0.92
> 12 weeks	0.79	0.37
≤35years	0.02	0.89
> 35years	1.46	0.83

Appendix 13.2 Inconsistency model for subgroups of FBG

FBG	chi2	Prob > chi2
≤ 12 weeks	5.88	0.75
> 12 weeks	1.58	0.21
overweight/obese	4.21	0.65
prediabetes	4.08	0.13

Appendix 14 Ranking in the subgroup of VAT and FBG

Subgroup analysis of VAT							
≤ 35 years (7studies, n=313)		>35years (13 studies, n=1151)		≤12weeks (14studies, 648)		>12weeks (6 studies, 816)	
Treatment	SUCRA	Treatment	SUCRA	Treatment	SUCRA	Treatment	SUCRA
HIIT	86.7	AT	96.5	HIIT	67	AT	94.9
AT	44.4	RT	56.9	AT	65.7	RT	60.4
CT	41.8	CT	51.4	CT	58.7	CT	43.8
CON	27.1	HIIT	43.1	RT	45.5	CON	0.9
-	-	CON	2.2	CON	13.1	-	-
Subgroup analysis of FBG							
≤12weeks (14studies, n=765)		>12weeks (11studies, n=1655)		overweight/obesity (10studies, n=654)		Prediabetes (12studies, n=1521)	
Treatment	Treatment	Treatment	SUCRA	Treatment	SUCRA	Treatment	SUCRA
MBE	100	MBE	93.7	RT	98.5	MBE	99.5
AT	61.9	RT	74.5	HIIT	64.7	HIIT	65
HIIT	56	AT	59.5	AT	37.5	AT	60.8
RT	43.4	CT	40.8	CON	29.9	RT	50
CT	32.8	HIIT	24.2	CT	19.2	CT	24.3
CON	5.9	CON	7.4	-	-	CON	0.4

Note. Three studies on prediabetes with overweight/obesity were excluded from the subgroup analysis of FBG.

Appendix 15 Network meta-analysis matrix for subgroup analysis of VAT and FBG

**Subgroup analysis of VAT**					
**≤12week**	HIIT	0.01 (-0.28,0.29)	0.03 (-0.39,0.45)	0.13 (-0.64,0.89)	0.26 (0.03,0.49)
	-0.01 (-0.29,0.28)	AT	0.02 (-0.37,0.41)	0.12 (-0.65,0.89)	0.25 (0.02,0.49)
	-0.03 (-0.45,0.39)	-0.02 (-0.41,0.37)	CT	0.09 (-0.74,0.93)	0.23 (-0.18,0.64)
	-0.13 (-0.89,0.64)	-0.12 (-0.89,0.65)	-0.09 (-0.93,0.74)	RT	0.14 (-0.59,0.86)
	**-0.26 (-0.49,-0.03)**	**-0.25 (-0.49,-0.02)**	-0.23 (-0.64,0.18)	-0.14 (-0.86,0.59)	CON
**＞12 week**	AT	0.22 (-0.20,0.64)	0.34 (0.06,0.63)	0.64 (0.44,0.83)	-
	-0.22 (-0.64,0.20)	RT	0.12 (-0.34,0.59)	0.42 (-0.00,0.83)	-
	**-0.34 (-0.63,-0.06)**	-0.12 (-0.59,0.34)	CT	0.29 (0.09,0.50)	-
	**-0.64 (-0.83,-0.44)**	-0.42 (-0.83,0.00)	**-0.29 (-0.50,-0.09)**	CON	-
**≤35years**	HIIT	1.00 (-1.04,3.04)	1.03 (-1.26,3.33)	1.26 (-0.17,2.70)	-
	-1.00 (-3.04,1.04)	AT	0.04 (-1.73,1.80)	0.27 (-1.19,1.72)	-
	-1.03 (-3.33,1.26)	-0.04 (-1.80,1.73)	CT	0.23 (-1.56,2.02)	-
	-1.26 (-2.70,0.17)	-0.27 (-1.72,1.19)	-0.23 (-2.02,1.56)	CON	-
**＞35years**	AT	0.23 (-0.14,0.60)	0.27 (0.01,0.53)	0.32 (0.03,0.60)	0.55 (0.38,0.72)
	-0.23 (-0.60,0.14)	RT	0.04 (-0.37,0.45)	0.08 (-0.36,0.52)	0.31 (-0.05,0.68)
	**-0.27 (-0.53,-0.01)**	-0.04 (-0.45,0.37)	CT	0.04 (-0.27,0.36)	0.28 (0.08,0.48)
	**-0.32 (-0.60,-0.03)**	-0.08 (-0.52,0.36)	-0.04 (-0.36,0.27)	HIIT	0.23 (-0.03,0.49)
	**-0.55 (-0.72,-0.38)**	-0.31 (-0.68,0.05)	**-0.28 (-0.48,-0.08)**	-0.23 (-0.49,0.03)	CON
**Subgroup analysis of FBG**					

**≤12week**	MBE	0.91 (0.38,1.44)	0.93 (0.38,1.49)	1.00 (0.43,1.56)	1.06 (0.52,1.59)	1.19 (0.71,1.67)
	**-0.91 (-1.44,-0.38)**	AT	0.02 (-0.27,0.32)	0.09 (-0.22,0.39)	0.15 (-0.15,0.45)	0.28 (0.06,0.50)
	**-0.93 (-1.49,-0.38)**	-0.02 (-0.32,0.27)	HIIT	0.06 (-0.33,0.45)	0.12 (-0.22,0.46)	0.26 (-0.03,0.54)
	**-1.00 (-1.56,-0.43)**	-0.09 (-0.39,0.22)	-0.06 (-0.45,0.33)	RT	0.06 (-0.32,0.44)	0.19 (-0.11,0.50)
	**-1.06 (-1.59,-0.52)**	-0.15 (-0.45,0.15)	-0.12 (-0.46,0.22)	-0.06 (-0.44,0.32)	CT	0.13 (-0.11,0.38)
	**-1.19 (-1.67,-0.71)**	**-0.28 (-0.50,-0.06)**	-0.26 (-0.54,0.03)	-0.19 (-0.50,0.11)	-0.13 (-0.38,0.11)	CON
**> 12week**	MBE	0.51 (-0.41,1.44)	0.63 (-0.29,1.54)	0.78 (-0.11,1.68)	0.94 (-0.07,1.95)	1.04 (0.17,1.91)
	-0.51 (-1.44,0.41)	RT	0.11 (-0.19,0.42)	0.27 (-0.12,0.65)	0.43 (-0.18,1.03)	0.53 (0.21,0.84)
	-0.63 (-1.54,0.29)	-0.11 (-0.42,0.19)	AT	0.16 (-0.20,0.52)	0.31 (-0.28,0.90)	0.41 (0.13,0.70)
	-0.78 (-1.68,0.11)	-0.27 (-0.65,0.12)	-0.16 (-0.52,0.20)	CT	0.16 (-0.40,0.72)	0.26 (0.04,0.48)
	-0.94 (-1.95,0.07)	-0.43 (-1.03,0.18)	-0.31 (-0.90,0.28)	-0.16 (-0.72,0.40)	HIIT	0.10 (-0.42,0.62)
	**-1.04 (-1.91,-0.17)**	**-0.53 (-0.84,-0.21)**	**-0.41 (-0.70,-0.13)**	**-0.26 (-0.48,-0.04)**	-0.10 (-0.62,0.42)	CON
**overweight/obese**	RT	0.33 (-0.02,0.69)	0.42 (0.04,0.80)	0.47 (0.12,0.82)	0.44 (0.12,0.76)	-
	-0.33 (-0.69,0.02)	HIIT	0.09 (-0.17,0.34)	0.14 (-0.06,0.33)	0.10 (-0.05,0.26)	-
	**-0.42 (-0.80,-0.04)**	-0.09 (-0.34,0.17)	AT	0.05 (-0.19,0.29)	0.02 (-0.20,0.24)	-
	**-0.47 (-0.82,-0.12)**	-0.14 (-0.33,0.06)	-0.05 (-0.29,0.19)	CT	-0.03 (-0.18,0.12)	-
	**-0.44 (-0.76,-0.12)**	-0.10 (-0.26,0.05)	-0.02 (-0.24,0.20)	0.03 (-0.12,0.18)	CON	-
**prediabetes**	MBE	0.58 (0.01,1.16)	0.65 (0.24,1.06)	0.71 (0.29,1.13)	0.92 (0.50,1.33)	1.16 (0.81,1.52)
	**-0.58 (-1.16,-0.01)**	HIIT	0.07 (-0.34,0.47)	0.12 (-0.33,0.58)	0.33 (-0.17,0.83)	0.58 (0.13,1.03)
	**-0.65 (-1.06,-0.24)**	-0.07 (-0.47,0.34)	AT	0.05 (-0.15,0.25)	0.26 (-0.03,0.56)	0.51 (0.31,0.71)

	**-0.71 (-1.13,-0.29)**	-0.12 (-0.58,0.33)	-0.05 (-0.25,0.15)	RT	0.21 (-0.10,0.52)	0.46 (0.23,0.68)
	**-0.92 (-1.33,-0.50)**	-0.33 (-0.83,0.17)	-0.26 (-0.56,0.03)	-0.21 (-0.52,0.10)	CT	0.25 (0.03,0.46)
	**-1.16 (-1.52,-0.81)**	**-0.58 (-1.03,-0.13)**	**-0.51 (-0.71,-0.31)**	**-0.46 (-0.68,-0.23)**	**-0.25 (-0.46,-0.03)**	CON

Note. AT, aerobic training; RT, resistance training; CT, combined aerobic and resistance training; HIIT, high-intensity interval training; MBE, mind-body exercise; CON, control group.

Appendix 16 Sensitivity analysis for VAT, FBG, and HbA1c

Appendix 16.1 Sensitivity analysis excluding extreme exercise dose

VAT		FBG		HbA1c	
Treatment	SUCRA	Treatment	SUCRA	Treatment	SUCRA
AT	82.3	MBE	100	MBE	73.6
HIIT	61.9	AT	57.9	AT	71
CT	57.9	RT	50.9	CT	63.8
RT	38.5	HIIT	47.8	RT	46.6
CON	9.5	CT	41.4	HIIT	36.3
-	-	CON	2	CON	8.8

Appendix 16.2 Sensitivity analysis excluding high risk of bias studies

VAT		FBG	
Treatment	SUCRA	Treatment	SUCRA
AT	83.2	MBE	100
HIIT	58.6	RT	62.9
CT	57.4	AT	59.2
RT	48.2	HIIT	39.9
CON	2.6	CT	37.6
-	-	CON	0.4
